# A Post-Surgical Retinal Progenitor Cell Niche is the Primary Source of Embryonic Eye Regrowth in *Xenopus laevis*

**DOI:** 10.64898/2026.05.03.722558

**Published:** 2026-05-10

**Authors:** Randolph L. Grell

**Affiliations:** School of Life Sciences, University of Nevada, Las Vegas, Las Vegas, NV 89154, USA

**Keywords:** eye regrowth, retinal progenitor cells, Xenopus laevis, lineage tracing, EosFP, optic vesicle, regeneration, stem cell niche, photoconversion

## Abstract

*Xenopus laevis* has recently emerged as a vital model for studying functional eye regrowth in pre-metamorphic tadpoles. Following eye removal surgery, tailbud embryos have been shown to regenerate a functionally complete eye within a 3–5 day period. While current studies have primarily focused on the signaling mechanisms required for this rapid regeneration, less is known about the specific stem cell populations and modes of regeneration employed by the embryo. In both the adult and tadpole, eye tissue regeneration can be facilitated through a combination of a pre-existing stem cell niche and the transdifferentiation of cells surrounding retinal or lens injuries, depending on the extent of the tissue removal. Notably, in the *Xenopus* eye regrowth assay, surgeries typically leave behind approximately 15% of the ocular tissue, indicating a post-surgical stem cell niche with potential for regeneration.

In this study, we explored the hypothesis that a residual retinal progenitor cell (RPC) niche is critical for the rapid eye regrowth observed in *Xenopus* tadpoles. By utilizing a photoconvertible protein, EosFP, which changes permanently from green to red fluorescence, we selectively marked retinal progenitor cells (RPCs) in the presumptive eye area with red fluorescence. We then carefully preserved a small population of these red-labeled RPCs within the post-surgical wound. This progenitor cell niche, comprising not only the red-labeled RPCs but also the surrounding cells, creates a unique signaling environment. This specialized microenvironment is crucial, as it may provide specific signals that dictate the developmental outcomes of the RPCs, effectively controlling their fate. Observations made throughout the regrowth process revealed that the eye predominantly regrew from this red-labeled RPC niche within three days, with all retinal layers comprising red-labeled cells. The regrown lens was observed to be composed of a mix of both cells outside the RPC lineage and RPC progeny. Of interest, we observed cells of the closing optic fissure and ventral retina incorporate progeny from cells outside the labeled RPC lineage. These findings support the notion that the primary mode of regeneration in pre-metamorphic *Xenopus* eye regrowth involves the use of a pre-existing stem cell niche, and may also involve transdifferentiation, thus providing new insights into the mechanisms of embryonic eye regrowth in *Xenopus laevis*.

## Introduction

Regeneration is the process by which cells, tissues, and organs repair and replace damaged or lost structures through the proliferation and subsequent differentiation of multipotent progenitor cell populations. Understanding the process of regeneration and its cellular and molecular origins has been a long-standing challenge in biology, and researchers have employed a variety of model organisms to understand the mechanisms of tissue regrowth. The process of eye regeneration is of particular interest due to the eye’s complexity and specialization. This is especially true for visually dependent organisms like humans, who lack the vigorous regenerative capacity seen in other vertebrate species. The process of eye regeneration has been studied in several model organisms, including planaria, newt, zebrafish, and *Xenopus* who display a great ability to regrow parts of their lens, cornea, retina, and whole eye in the case of planaria and newt (LoCascio et al., 2017; Reddien, 2021; Islam et al., 2014; Tsonis & Del-Rio Tsonis, 2004; Wan & Goldman, 2016; Tseng, 2017). Each organism has its own strengths and weaknesses as a model of regrowth. Planaria have the extraordinary ability to regenerate all parts of their body after injury (Mannini et al., 2004; Inoue et al., 2004; Hill & Petersen, 2018; Reddien, 2018). However, this exotic form of regeneration can also produce genetically identical clones of the flatworm when dissected into hundreds of pieces (Lobo et al., 2012), potentially leaving a large mechanistic gap when trying to develop regenerative strategies for human regeneration. The newt, particularly the axolotl, can completely regrow most of its limbs and organs throughout life, but again lacks a regenerative profile that more closely resembles the limited capacity seen in humans (Wells et al., 2021; Jensen et al., 2021; Yandulskaya et al., 2022). *Xenopus laevis*, however, has recently emerged as a vertebrate model that has both a large capacity for regrowth in the eye during the pre-metamorphic tadpole stage and a limited capacity for regrowth during the adult phase of its life cycle (Vergara & Del Rio-Tsonis, 2009; Ueda et al., 2012; Lee et al., 2013; Hamilton & Henry, 2016; Kha et al., 2018a). This offers a unique opportunity to identify the genetic, transcriptomic, and epigenetic changes that occur from a highly regenerative phase to a limited regenerative phase within the same closely related vertebrate organism.

Previous research into eye regrowth has focused both on the adult *Xenopus* frog as well as pre-metamorphic tadpole eye, both of which share similar cellular mechanisms of tissue regeneration within the eye (Vergara & Del Rio-Tsonis, 2009; Ueda et al., 2012; Lee et al., 2013; Hamilton & Henry, 2016; Kha et al., 2018a). It has been demonstrated that in adult post-metamorphic *Xenopus*, ocular structures such as the lens and retina can regenerate after injury or removal (Yoshii et al., 2007; Araki, 2014), while the cornea is adept at maintaining tissue homeostasis and wound healing (Adil et al., 2019). These three tissues exhibit distinct mechanisms of recovery, each related to different sources of progenitor cells required for tissue regeneration or maintenance. For instance, depending on the species of *Xenopus*, the retina is able to repair itself from the major contribution of cells from the retinal pigmented epithelium (RPE) in the central portions of the retina or in the periphery from an existing adult stem cell niche in the ciliary marginal zone (CMZ). In *Xenopus laevis*, when regions of the central retina are damaged, cells of the RPE break away, migrate, and accumulate at the inner limiting membrane of the retina through the process of epithelial-mesenchymal transfer (Yoshii et al., 2007). This process of transdifferentiation permits RPE cells to take on a less differentiated progenitor cell state to increase in number and eventually differentiate into cells of the mature regrown retina (Yoshii et al., 2007). In *Xenopus tropicalis*, the peripheral portions of the retina are more amenable to regrowth from differentiation of an existing adult stem cell niche in the ciliary marginal zone of the eye (Miyake & Araki, 2014). The lens in adult *Xenopus*, while once thought not to regenerate after metamorphosis (Freeman, 1963), has been recently shown to regrow in the adult frog. When the lens is removed, the source of this regeneration is thought to be from the expansion and differentiation of remaining lens epithelial cells within the eye (Yoshii et al., 2007). Corneal tissue utilizes a mode of regeneration that relies on a remaining adult stem cell niche within the limbal tissue, specifically located in the basal layer of the peripheral cornea, comprised of Corneal Epithelial Stem Cells (CESCs) (Adil et al., 2019).

These modes of regeneration also are evident in the pre-metamorphic tadpole stage where the mature tadpole eye can regrow a damaged or removed retina via a combination of transdifferentiation of the RPE and differentiation of an existing stem cell niche within the CMZ (Araki, 2007; Martinez-De Luna et al., 2011; Parain et al., 2023). The lens may regrow through transdifferentiation of corneal cells (Waggoner, 1973; Reeve & Wild, 1978; Bosco et al., 1981; Bosco et al., 1993; Gargioli et al., 2008; Hamilton & Henry, 2016), and the cornea may replenish or heal from injury through the transdifferentiation of limbal stem cells (Perry et al., 2013; Sonam et al., 2020; Sonam et al., 2022). These examples demonstrate two prominent modes of regeneration active in both the adult and tadpole stages of the *Xenopus* life cycle.

*Xenopus* eye regrowth has also been seen at earlier embryonic stages in development. In the context of retinotectal map plasticity Berman and Hunt showed that when 40–60% of the st. 25/26 optic cup, st. 31/32 optic vesicle, and st. 38 larval eye was removed the eye grew back at a diminished or normal size (Berman & Hunt, 1975). Moreover, these regrown eyes were shown to form physiologically active synapses with cells in the optic tectum. This group postulated that the source of the regenerating eye tissue may have originated from the optic stalk during these points in development. More recently our lab has shown that when approximately 83% of the st. 27 optic vesicle is removed the eye regrew within a 5-day period, maintained gross functionality in a swimming behavioral assay, and that the critical period for this rapid regrowth of eye tissue peaked at st.27 in development (Kha et al., 2018a; 2018b). Furthermore, it has been demonstrated that the regrown eye develops all cell types found in the mature retina, albeit with an initial 24-hour delay. During this period, retinal progenitor cells undergo rapid proliferation, leading to subsequent differentiation and retinal patterning (Kha et al., 2019). This process continues for two more days post-surgery, at which point the size and pattern of the regenerated eye closely resemble those of a normal, unoperated eye at a comparable developmental stage (Kha et al., 2019).

Despite the progress made in understanding the mechanisms of embryonic eye regeneration in *Xenopus laevis*, the exact source of the regenerating cells remains unknown. The previous studies of st. 27 eye regrowth have shown that on average 83% of the optic vesicle is able to be removed. The nature of this surgical procedure inherently leaves a small population (17% of the eye) of rapidly dividing retinal progenitor cells within the wound region, which doubles in population approximately every 8.6 hours during endogenous eye development (Rapaport, 2006). This remnant pool of retinal progenitor cells, with their rapid division rate, may provide a post-surgical stem cell niche capable of proliferating and differentiating to regrow the entirety of the eye.

Furthermore, the regrowth of eye structures occurs during a developmental stage when mature components such as the retinal pigment epithelium (RPE), retinal vascular membrane, ciliary marginal zone (CMZ), and limbal stem cells—which typically contribute to the regeneration of tadpole or adult eyes through transdifferentiation—are not yet present. This leaves a gap in our knowledge regarding the mode of regeneration occurring within the wound region of the extirpated embryonic *Xenopus* eye. Does eye regrowth originate from an existing proliferative stem cell niche left over after eye removal, or does it result from the transdifferentiation of surrounding cells—such as those from the developing brain, optic stalk, olfactory placode, or epidermis—in response to disrupted signaling or the mechanical removal of the eye?

Based on these observations, we formed the hypothesis that a proliferative stem cell niche, created as a byproduct of stage 27 eye removal surgery, contributes to the rapid regeneration observed five days post-surgery. We addressed our hypothesis using a novel photoconvertible protein, EosFP, to specifically label the remnant eye progenitor cells left over from an eye removal surgery and traced their lineage through the regenerative process. EosFP is a protein originally found in the stony coral *Lobophyllia hemprichii*, which permanently changes its spectral properties from a green to a red fluorescing protein upon exposure to near ultraviolet light. Using EosFP in conjunction with confocal laser microscopy allowed us to label and track the post-surgical RPC niche *in vivo*, enabling us to determine if these cells were capable of regrowing the removed eye.

## Methods and Materials

### Embryo Selection Culture, and Injection

Embryos were obtained via *in vitro* fertilization and raised in 0.1X Marc’s Modified Ringer (MMR, 0.1 M NaCl, 2.0 mM KCl, 1 mM MgSO4, 2 mM CaCl2, 5 mM HEPES, pH 7.8) medium (Sive et al., 2000). *Xenopus* embryos were de-jellied using a 3% cysteine solution in deionized water for 5 min 1-hour post-fertilization in preparation for microinjection and manipulation (Sive et al., 2007). Embryos were then incubated at 14°C in .1x MMR until the second cleavage at the 4-cell stage. Embryos were selected for injection at the four-cell stage as follows: At the 2-cell stage embryos that have their first cleavage furrow bisecting the lightly pigmented portion of the animal hemisphere were obtained. These embryos at the 4-cell stage were then injected only if the cleavage furrow bisects the embryo into dorsal and ventral hemispheres of equal sizes as previously outlined in Klein (1987), Moody (1987a, 1987b), and Moody and Kline (1990). At this point the dorsal blastomere can be injected under a 3% Ficoll solution with previously prepared mRNA coding for the photoconvertible EosFP at a concentration 0.5–1 μg/μL with a total injection volume of no more than 2–4nL to prevent cytotoxicity as outlined in (Chernet et al., 2012). mRNA injections were directed towards the central portion of the dorsal blastomere. This will aide in Eos mRNA being equally partitioned out to daughter cells as the blastomere goes through subsequent cleavages. After injection embryos were washed three times for 5 minutes in 0.1x MMR then placed into a new 60×15mm petri dish of 0.1x MMR at 14°C for 1 day. Embryos were allowed to develop to Nieuwkoop and Faber stage 15 (Nieuwkoop and Faber, 1994) and were analyzed under a V20 epifluorescence microscope with a TRITC filter then selected for photoconversion based on their expression pattern of the green form of EosFP. These embryos (n= 100) were then taken to a confocal microscope for photoconversion before the close of stage 15 and separated into different experimental groups for assessing the specificity of photoconversion to RPCs, eye removal quantification, and to assess the role of RPCs in tadpole eye regrowth (Grell, in preparation; Grell, 2026).

### Photoconversion

Stage 15 embryos were then subject to photoconversion by way of a Nikon A1R upright scanning confocal microscope equipped with a 408nm wavelength laser. All photoconversions were done through a 10x water dipping objective with a laser intensity of 65% with an imaging protocol that would image the embryo in the green channel before photoconversion then photoconvert with the 408nm laser for 5sec followed by 5sec of no laser. To specifically direct the photoconversion to our cells of interest within the stage 15 eye field, the freehand Region of Interest (ROI) tool on the Nikon A1R was employed to confine the microscope’s UV laser scan area. A region on the anterior neural plate, known to host the eye field, was selected, as previously demonstrated by Zuber et al. (2003) and (Grell, 2026). The UV laser was then assigned to scan only this area to limit our photoconversion to cells of the presumptive eye with 100% scan speed on a resonant scanner to decrease phototoxic effects on cells as well as decrease labeling outside the designated region of interest which is associated with lower scan speeds and higher laser intensities. This was repeated until red photoconverted cells were distinctly seen in our software defined region of interest. Immediately post photo conversion both the green and the red laser were used to image and track the ongoing conversion of green to red fluorescent protein and its position on the neural plate. Once completed a final image 200μm through the z-plane was made in the red, green, and brightfield channels to accurately locate the final photoconversion on the anterior neural plate of the embryo. Once imaged, embryos were transferred to fresh 0.1x MMR and placed in a 14°C incubator until they reached developmental stage 27. At st. 27, each photoconverted embryo was evaluated for the presence of red Eos protein filling the optic vesicle area, ensuring it was confined solely within the eye. These selected embryos were then subjected to confocal microscopy imaging prior to eye removal surgery. The overall success rate of green EosFP expression in the anterior neural plate following D1 dorsal blastomere injection was approximately 90%. However, the success rate of photoconversions within the eye field was limited to 10%, contingent upon the clarity of the embryo’s surrounding physical features, such as the neural ridge’s distinctiveness, the closing neural tube, and any signs of an optic depression forming on the anterior neural plate. Of the embryos that exhibited exclusive red EosFP labeling within the eye field, an average of 7% survived the entire experimental procedure, which included imaging, eye removal surgery, and subsequent re-imaging at 2 hours post-surgery (hps) and then every 12 hps until stage 42. This survival rate was influenced by various factors, including the overall health of the embryos from the frog mother, the tailbud embryos’ tendency to swim and occasionally collide with the air-water interface with an open wound leading to death, and the handling of the fragile embryos post-surgery.

### Labeling of RPCs in a Single Neural Ectoderm Layer

To investigate whether retinal progenitor cells (RPCs) can be selectively labeled in a single cell layer without affecting the underlying mesodermal tissue, we utilized photoconversion at Stage 15 of development. At this stage, RPCs were labeled with the green form of EosFP within the eye field and subsequently photoconverted to red. Following the photoconversion, embryos were fixed with a 4% formaldehyde solution for 2 hours at room temperature. Horizontal sections of these embryos, at a thickness of 60 μm, were prepared on a vibratome. This step was designed to facilitate the identification and localization of red-labeled cells within the neuroectoderm. Additionally, to confirm the specific localization of red-labeled RPCs in the neuroectoderm and to ensure they were not present in the underlying mesoderm, optical sections were obtained using a multiphoton microscope (n=8 embryos). This imaging targeted the eye field and extended to a depth of 200 μm, with sections at 1 μm intervals.

### Labeling of RPCs in the Optic Vesicle

To assess the labeling of the entire RPC population in the optic vesicle, we photoconverted the left eye fields (as described in Grell, in preparation) of n=5 embryos initially labeled with green EosFP to the red form of EosFP. The embryos were allowed to develop until they reached embryonic st. 27. The embryos were then imaged to identify the location of the red-labeled RPCs on the surface of the embryo. Subsequently, they were fixed in a 4% formaldehyde solution at room temperature for 2 hours. After fixation, the embryos were embedded in a 6% Aragose solution which was allowed to solidify, then underwent sectioning on a vibratome. Transverse sections with a thickness of 60 μm were prepared and imaged to evaluate the distribution and extent of red-labeled RPCs within the optic vesicle (Figure 3). All live embryos were incubated in a .02% Phenylthiourea (PTU) at 22 °C to prevent melanin synthesis from occurring. This prevented pigmentation in the retinal pigmented epithelium (RPE) allowing for live imaging of cells through regrowth and the subsequent imaging of the transverse sections after fixation and sectioning.

### Eye Removal Surgery

Eye removal surgeries were based on published surgical techniques (Kha et al., 2018a). Embryos at Nieuwkoop and Faber stage 27 were anaesthetized with MS222 (Sigma). Using surgical forceps drawn to a fine point (Dumont No. 5) a cut was made in the skin at one corner of the left optic vesicle. The cut was continued to circumscribe the outline of raised eye at which point the eye would lift out of the wound region intact and with little effort. After surgery, embryos were washed of MS222 and transferred into 0.1x MMR. Embryos were placed in a 14 °C incubator for 3 days and allowed to recover.

To determine the amount of tissue left over from the eye removal surgery a group of n=30 embryos had their eyes surgically removed at st. 27 and were immediately fixed in MEMFA. Tailbud embryos were then processed for tissue sectioning and prepared according to (Blackiston et al., 2010). Tailbud embryos were embedded in 6% agarose and sectioned into 60 μm slices using a Leica vt1000s vibratome through the transverse plane of the head region. Sections were then subjected to immunohistochemistry as outlined in (Kha et al., 2018a) where Xen1 primary antibodies were used to detect the pan-neural Xen1 protein in neurons within the remaining portions of the removed eye (Figure 4). The remaining portions of the eye for each section (n=3 sections per eye) were taken as percentage of the total area of the right contralateral unoperated control eye and an overall average of remaining Xen1 eye tissue was calculated for the 30 tailbud embryos. To indicate how surgical technique influenced the quality of regrowth and compare it to the average amount of tissue removed, a regrowth index was calculated as previously described (Kha et al., 2018a, Tseng et al., 2010). The resulting value, known as the Regeneration Index (RI), ranges from 0 to 300, with 0 indicating no regeneration in any of the individuals in the group and 300 indicating complete regeneration in all individuals in a dish.

### Assessment of Stem Cells Involved in Eye Regrowth

To determine if a population of remnant retina progenitor cells has the capacity to regrow a surgically removed eye, mRNA that codes for the photoconvertible protein EosFP was injected at the 4-cell stage in development into the dorsal blastomere. Embryos were allowed to develop to st. 15 where embryos with a green-fluorescent expression pattern found restricted to the nervous system were selected for photoconversion. At stage 15 in development, it has previously been shown that the eye field is contained within the anterior neural plate and is composed of a monolayer of retinal progenitor cells (Zuber et al., 2003). Photoconverting the eye field at this point ensures that all cell types in the future eye will be labeled with our red lineage tracer in contrast to the surrounding green labeled nervous system.

Labeled embryos grown to st. 27 and which contained red lineage tracer only within their eye were imaged, anesthetized, and subjected to an eye removal surgery of the eye containing the red photoconverted protein. To determine the location of the red labeled retinal progenitor cell niche optical sections were taken through the depth of the wound region in the parasagittal plane post-surgery (n=15). This series of images through the depth of the eye were used to create a three-dimensional image indicating where the remnant retinal progenitor cell niche is within each of the operated tailbud embryos. Embryos were then gently washed in 0.1X MMR then placed in a fresh dish of 0.1X MMR and incubated at 14°C.

### Imaging Regrowth

Labeled and operated embryos were then imaged through the process of regrowth at specific time points. All images were generated on a Nikon 1R scanning confocal microscope starting immediately after the initial eye surgery and then again at 2 hps, 14 hps, and then every 12 hps until the experimental embryos reached Nieuwkoop and Faber stage 40 (Nieuwkoop & Faber, 1994), where the cells of the eye have started to differentiate and have stratified to their normal retinal and ocular anatomy (n=15). Of the 15 experimental embryos that survived from surgery to st. 40, 5 embryos were chosen to further mature until st. 48 to assess the location of the red converted lineage tracer throughout the depth of the eye. These embryos were chosen based on the high intensity and coverage of the red label specifically within the eye, facilitating tracking of retinal cells to the later embryonic stage. To accomplish this, multiphoton microscopy was used to take optical sections in both the red and green channels at 5 μm intervals starting from the lateral most portion of the eye (top of the lens) and finishing when the entire thickness of the eye has been imaged (most medial portion of the eye). The multiphoton imaging was done *in vivo* to preserve the intensity of the red fluorescent signal that tends to decrease at these later developmental stages and with formaldehyde fixation. The acquired images were subsequently analyzed and processed using Nikon Imaging Software V5.21.00. An additional n=5 experimental tadpoles were generated and imaged at less frequent time points to preserve the integrity of the lineage tracer for multiphoton imaging after st. 42.

### Identifying Photoconverted RPCs

To accurately determine the presence of the red photoconverted form of EosFP in cells, we analyzed images captured at various time points across red, green, and combined channels. The intensity levels in both red and green channels were quantitatively assessed using the software associated with the confocal microscope (Figure 6). This analysis enabled us to calculate a red:green intensity ratio, providing a valuable metric for identifying cells containing the photoconverted protein and estimating the relative concentration of red:green EosFP within a cell at any given time. Additionally, the labeled cells were qualitatively categorized by viewing them in both red and green channels. This dual-channel visualization was employed to observe the dilution of the red lineage tracer relative to the green form of the protein over time, particularly as cell proliferation occurs.

### Determining the Cell Populations Involved in Lens Regrowth

Our initial data indicated that lens regrowth involved both red and green labeled cells. To investigate whether st. 27 epidermal cells specifically contribute to lens regrowth, we allowed green Eos labeled tadpoles to develop until st. 27. These embryos were selected for their green optic vesicles and green-labeled skin surrounding the eye. We then photoconverted the epidermal cells surrounding the ventral half of the eye, creating a semi-circle of red around the bottom of, but not in the eye itself (Figure 13). The tadpoles were imaged, the green eye was removed, and subsequent imaging was performed post-surgery, 2 hps, 18 hps, and at st. 42 in development. During this period, we tracked the location of the red cells throughout the regrowth process. At st. 42, the regrown eyes were subjected to multiphoton imaging to examine their internal anatomy. This procedure was conducted with a group of n=10 different tadpoles.

## Results

### Labeled RPCs are Labeled as a Single Neural Ectoderm Layer

**Figure 1:**
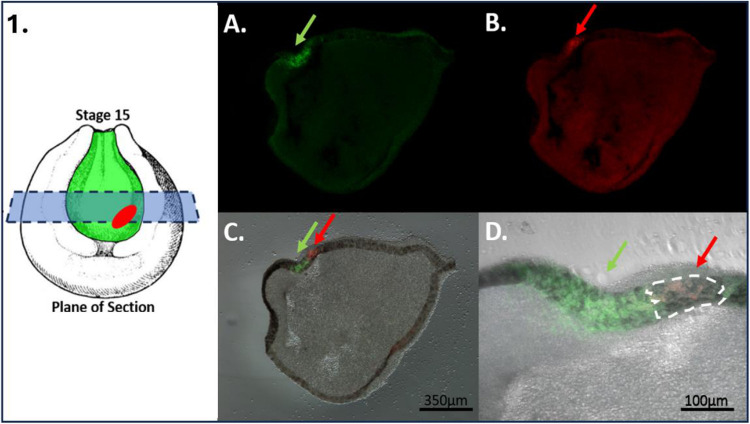
Eye Field Labeling Constrained to the Neural Ectoderm (Physical Sections). To determine if our lineage tracer is constrained to the neural ectoderm and does not label the underlying mesoderm, we photoconverted cells in the eye field from green to red in st. 15 embryos and sectioned them in the horizontal plane (n=5). Panel **(1)** indicates the plane of section. **(A)** Indicates the green non-photoconverted neural plate, with the green arrow pointing to the location of the green EosFP. **(B)** Shows the location of the photoconverted red RPCs (red arrow indicates the location of red EosFP in RPCs). **(C and D)** Display both the red-photoconverted RPCs in relation to the green neural plate. Panels B, C, and D illustrate that red cells are only found in the most superficial cell layer of the embryo and not in the underlying mesoderm. Drawing modified from Nieuwkoop and Faber (1994).

**Figure 2:**
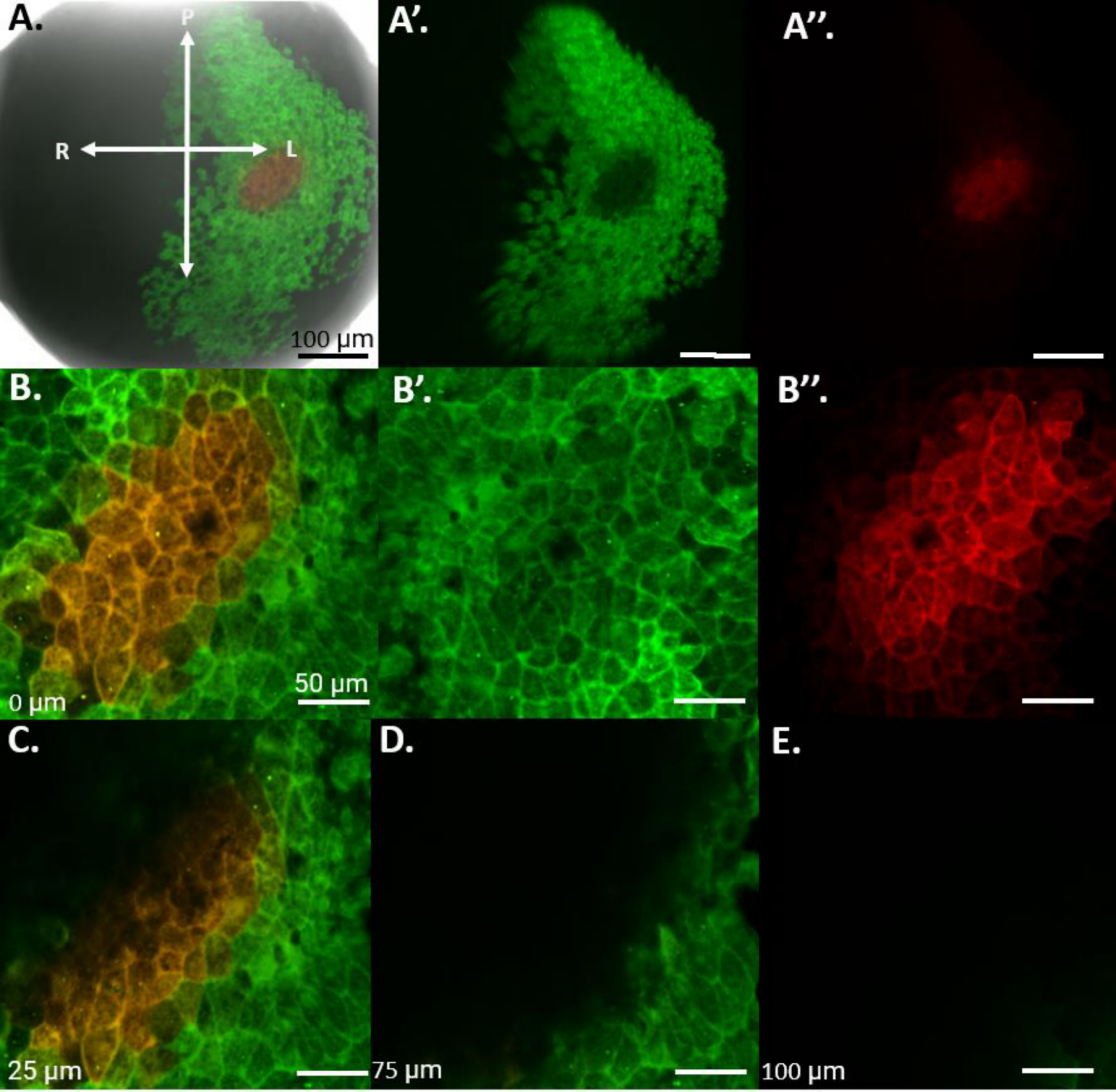
Eye Field Labeling Constrained to the Neural Ectoderm (Optical Sections). To further confirm that the mesoderm did not contain any red-labeled cells, n=5 embryos were photoconverted red in the eye field at st. 15. Optical sections were taken using a multiphoton microscope, starting from the embryo’s surface and extending to a depth of 150 μm at 1 μm increments. **(A)** Displays a confocal image of a st. 15 embryo with the left eye field photoconverted red, using a 10x objective lens. (A’ and A” indicate the green and red channels respectively. **(B)** Shows the same embryonic eye field under a 25x objective, with panels B’ and B” showing the green and red channels only. Panels **(C, D, E)** present the same embryo, but at depths of 25 μm, 75 μm, and 100 μm respectively. Across all analyzed embryos, none exhibited red-labeled cells beneath the superficial layer of neural ectoderm. The depths of 0 μm, 25 μm, 75 μm, and 100 μm indicate the section depth at which each image was taken. The orientations are indicated as R= right, L= left, P= posterior, A= anterior.

To determine whether retinal progenitor cells (RPCs) are labeled as a single cell layer while avoiding labeling of the underlying mesodermal tissue, we labeled n=5 *Xenopus* embryos at stage 15 of development with the red form of EosFP within the eye field. We then fixed the embryos and took horizontal sections at 60 μm thickness to locate the red-labeled cells. The results, as seen in Figure 1 show a 60 μm horizontal section of a Stage 15 embryo, highlighting the location of the green-labeled neural plate in relation to the labeled red region of the eye field. The red cells were found to reside within a single superficial layer of the neuroectoderm surrounded by green labeled cells. No red or green-labeled cells were observed within the cell layers underneath the neural plate. To further confirm the location of the red-labeled RPCs in the neuroectoderm and not the underlying mesoderm, we took optical sections using a multiphoton microscope through the eye field, down to a depth of 200 μm at 1 μm increments (Figure 2). In our analysis, the red-labeled RPCs were observed only within the first 20 μm of the embryo’s depth. This observation aligns with our estimates of the neural plate’s depth, which we determined to be 20 μm based on both physical and optical sectioning of stage 15 embryos labeled in the neural plate. Furthermore, in the optical sectioning of Stage 15 embryos, none of the n=5 embryos exhibited green-labeled cells, indicating that our mRNA injections specifically targeted the neural plate without affecting the underlying mesoderm. This specificity ensures that our red lineage tracer exclusively labeled the eye field and the future eye.

### Red-Labeled RPCs Found Throughout the Optic Vesicle

**Figure 3:**
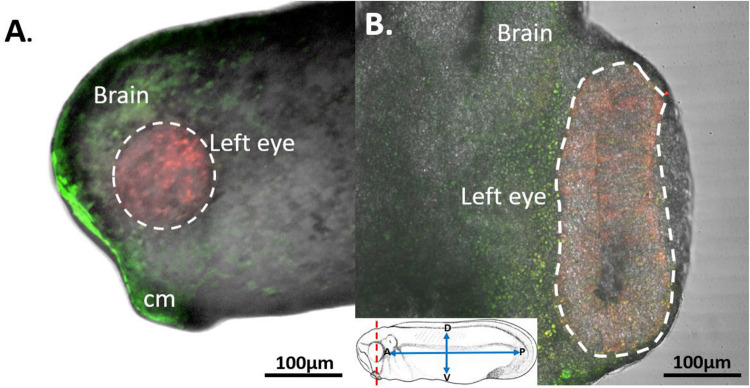
Photoconverted Cells Found Only Within the Developing Eye. To determine the extent and specificity of RPC labeling, embryos were photoconverted at st. 15 in the eye field and subsequently allowed to grow to st. 27, where the developing optic vesicle becomes evident. **(A)** Illustrates the location of red-labeled cells confined within the region of the left optic vesicle (dashed circle). Surrounding the eye, green-labeled cells are evident in the epidermis along the side of the tailbud embryo. Panel **(B)** presents a transverse section through the same embryo at the level of the red-labeled optic vesicle (dashed outline). Red-labeled RPCs are found throughout the depth of the optic vesicle, while green-labeled cells are located outside the eye. No red cells are present in the brain or in mesodermally derived tissues surrounding the developing eye. The inset provides a schematic of a st. 27 embryo with labeled anatomical orientation. The red dashed line indicates the plane of section through the embryo. D= dorsal, V= ventral, P= posterior, A= anterior, cm= cement gland. Drawing modified from Nieuwkoop and Faber (1994).

With the aim of establishing the specificity of our st. 15 eye field labeling, we allowed a group of n=5 embryos to grow to st. 27, a developmental stage when the optic vesicle is clearly evident. We initially imaged whole tailbud embryos and confirmed that the red-labeled cells were confined solely to the region of the st. 27 optic vesicle, with no detection of red cells in the surrounding epidermis (n=5). Subsequently, the imaged embryos were fixed and sectioned to examine the internal distribution of the red-labeled cells within the optic vesicle. These red-labeled cells were observed to extend throughout the entire depth of the invaginating optic vesicle. Importantly, the red-labeled cells were restricted to the developing eye regions, with no photoconverted cells found in the brain, optic stalk, or mesodermally derived tissues surrounding the eye. This demonstrates the specificity of our photoconversion labeling to the presumptive eye (Figure 3).

### Quantification of Post-Surgical RPC Niche

**Figure 4:**
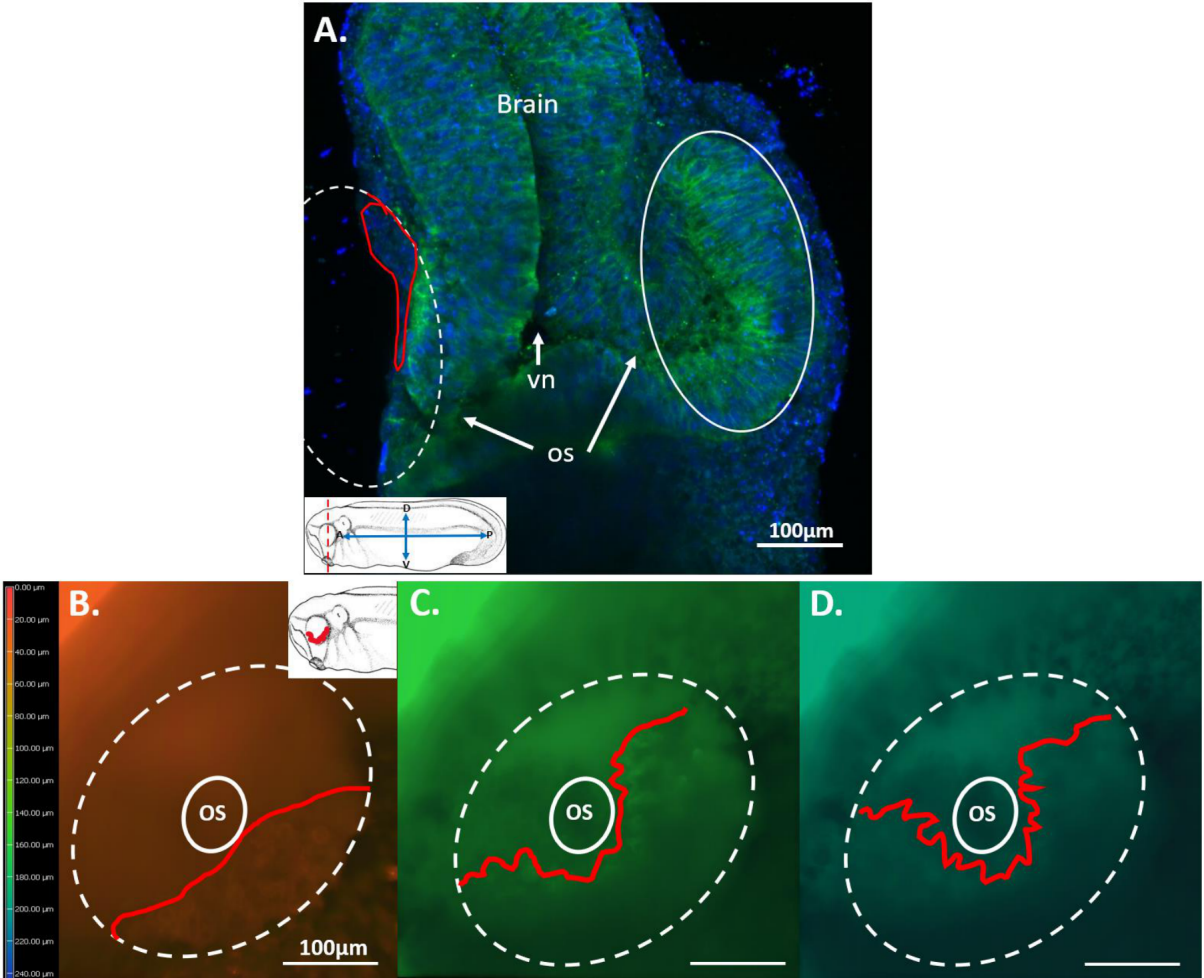
Quantifying the Remaining RPC Population After Eye Removal Surgery. Panel **(A)** displays a 60 μm transverse section through the head and eye region of a st. 27 tadpole after undergoing eye removal surgery on the right eye, with the wound region indicated by a dashed oval. The tissue was immunolabeled with the pan-neural marker Xen1, which appears green in the brain and eye tissue. Blue dots represent DAPI-labeled cell nuclei. The red outline within the wound region highlights the remaining Xen1-immunolabeled eye tissue post-surgery, indicating the residual pool of post-surgical RPCs. Panels **(B-C)** Illustrates the location of RPCs remaining after an eye removal surgery down the depth of the wound bed in a st. 27 tailbud embryo labeled with EosFP. Colors indicate the depth of the spherical RPCs within the wound and can be used to calculate the volume of the post-surgical RPC niche in a living embryo. Red line indicates the leading edge of in focus RPCs where the measurement was taken from in each image. The cells visible within the red line in panel **(B)** are at the level of the surrounding epidermis at 0 μm (see color scale in Panel B). Panel **(C)** indicates the depth of the RPCs at 120 μm depth while panel **(D)** shows the RPCs down to a depth of 200 μm before ending at the optic stalk. The red dashed line in the inset shows the plane of section through a st. 27 tailbud embryo. The solid white oval represents the unoperated control eye. Annotations: vn= ventricle, os= optic stalk, D= dorsal, V= ventral, P= posterior, A= anterior. Drawing modified from Nieuwkoop and Faber (1994).

To quantify the amount of RPCs remaining within the wound region after a st. 27 eye removal surgery, we utilized a group of n=30 tadpoles. The area of the removed eye was calculated as a percentage of the contralateral, unoperated control eye. Our analysis revealed that approximately 87% of the developing eye is typically removed during st. 27 eye removal surgery, leaving 13% of the eye behind on average. This finding is consistent with a calculated regenerative index of 289, derived from an analysis of n=50 tadpoles, and aligns with the extent of tissue removal reported in Kha et al., 2018a. Figure 4 depicts a sectioned st. 27 tailbud embryo, illustrating a comparison between the head region with the right eye removed and the total area of tissue in the unoperated eye.

**Figure 5:**
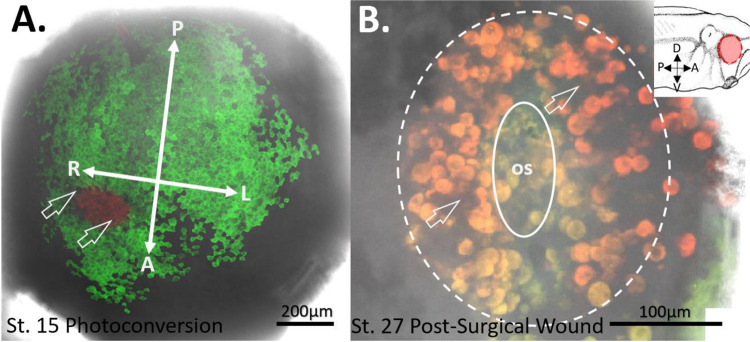
Distribution and Anatomy of Red-Labeled RPCs in the Wound Region Post-Eye Removal Surgery. While not used for quantifying RPCs within the post-surgical wound, optical sections down the depth of the wound reveal the distribution and anatomy of RPCs after surgery. Panel **(A)** displays a st. 15 embryo where the right eye field has been labeled red. White-outlined arrowheads denote RPCs within the eye field that lack green or red EosFP within the cells. Panel **(B)** presents an expanded view of the same tadpole at st. 27 post-eye removal. Optical sections have been merged into a single image. A dashed oval indicates the location of the wound, while a solid oval highlights the region where the optic stalk leads into the brain. White-outlined arrowheads point to possible progeny of the unlabeled cells seen in Panel A. Round red cells represent RPCs, while green cells within the optic stalk are situated outside the eye cell lineage. The diagram inserted into Panel B illustrates the orientation of the embryo for imaging, with the red oval denoting the region where the eye was removed. Annotations: os= optic stalk, D= dorsal, V= ventral, P= posterior, A= anterior. Drawing modified from Nieuwkoop and Faber (1994).

Although not directly utilized for quantification in eye removal surgeries, confocal images of red-labeled RPCs within the wound region post-surgery were captured (refer to Figure 5 Panel B, and Figures 9, 10, and 11, Panels C). These images were taken at 5 μm increments from the surface of the epidermis down into the wound, reaching a depth of up to 100 μm. Red-labeled. RPCs were consistently found beneath the plane of the surgical incision. However, the distribution of these cells varied, likely due to the stochastic nature of the optic cup removal process, despite the consistency of the surgical procedure and cutting technique.

The tissue remaining within the wound region was predominantly located in the ventral portions of the eye, extending dorsally on either side of the wound and towards the embryo’s midline to a depth of 100 μm, where a green-labeled optic stalk was observed. Post-surgery progenitor cell morphology revealed spherical cells with well-defined and unlabeled nuclei, their cell bodies measuring between 10–17 μm in diameter. A merged image of the wound region is presented in Figure 5. A dashed oval represents the region where the optic vesicle was removed, while the inner dashed circle denotes the beginning of the optic stalk. Red and diluted red (orange-colored) retinal progenitor cells (RPCs) exhibit a spherical morphology with an unlabeled nucleus in the center and are found throughout the wound bed. These red cells are also observed outside the dashed oval and are part of the remaining optic vesicle that was not removed during surgery. Green circular cells are located within the region of the optic stalk (solid oval line in Figure 5, Panel B), whereas green non-photoconverted cells were found outside the eye in the olfactory placode on the anterior-ventral end of the embryo. Unlabeled cells, containing neither green nor red EosFP, were identified throughout the wound bed, with two areas of these cells being indicated by white arrows in Panel B. Patches of dark to black unlabeled cells can also be seen in Figure 5 Panel A, surrounding the periphery of the red-labeled region, with at least one encroaching within the red-labeled area. Two white arrows in Figure 5 Panel A point to the location of two such cells. The unlabeled cells in Figure 5 Panel B might be progeny of these cells, given their location directly on the border of the eye field, or they could be descendants of some other adjacent unlabeled cell population. Embryos displaying this interspersed non-labeling were excluded from the n=10 used for tracking RPC regrowth.

While optical sectioning with the confocal microscope provided insights into the location and extent of these cells within the wound, future studies might benefit from incorporating multiphoton microscopy. This technique could offer enhanced single-cell resolution at depth, potentially providing a more detailed method for quantifying labeled RPCs within the wound region of each operated animal *in vivo*. Such an approach would greatly elucidate the impact of the number and distribution of cells remaining post-surgery on the quality and extent of regeneration observed for various surgical outcomes.

### High Red to Green Eos Ratio Used to Track RPCs

**Figure 6:**
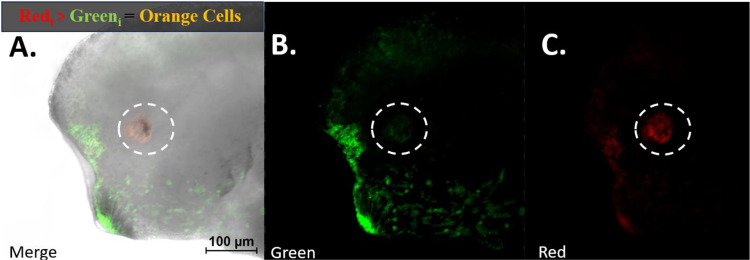
High Red to Green Eos Ratio Used to Track RPCs. Using the Nikon imaging software associated with the confocal microscope, we calculated the intensity of the red and green fluorescent channels in the region of the regrowing eye. This analysis enabled us to form a ratio of red:green EosFP intensity. This ratio is initially high immediately after photoconversion but gradually narrows as the red tracer is diluted due to cell proliferation and the continued translation of green Eos. Despite the narrowing ratio, cells are still visible in the combined red and green channels, displaying shades ranging from red to orange, then yellow to yellow-green. These color transitions serve as an indirect marker of cell proliferation. This approach remains effective for up to five days post-surgery in the regrowing eye. Panel **(A)** presents a merged image of green and red channels where the intensity of red (22.797 arbitrary units (A.U).) is greater than that of green (3.639 A.U.) within the regrowing eye, resulting in a red-orange hue. Panel **(B)** displays the green channel and the region where the intensity measurement was made, encircled by a dashed line. Panel **(C)** illustrates the red form of Eos and its associated region where the intensity measurement was made. When the ratio is calculated and is greater than 1, the cells are likely to have been photoconverted. This method helps eliminate the confusion caused by spontaneous red Eos formation over time, particularly in areas of the tadpole initially having high concentrations of green EosFP, which leads to higher background levels of spontaneously converted EosFP in their tissue. This is evident in Panel C, where the cement gland and olfactory bulb appear lightly red due to high concentrations of EosFP, yet their red:green ratio is less than 1, indicating they have not undergone laser photoconversion. These cells appear bright green in the merged image seen in Panel A.

To discern the location of the red photoconverted form of EosFP in cells and separate it from background noise, including spontaneous Eos conversion over time, we analyzed images at various time points across red, green, and combined red-green channels. We outlined the region initially photoconverted and calculated a red:green signal intensity ratio for this area (Figure 6). Throughout our time course analysis, this metric supplemented our qualitative assessment of the color of the photoconverted labeled cells.

We found that following photoconversion, the red EosFP always exhibited a higher concentration compared to its green counterpart. This predominance of red over green was consistent during the 5-day regrowth. Nonetheless, translation of the green form of EosFP still occurred within this timeframe from remaining mRNA within the cell. Consequently, the red:green Eos ratio gradually narrowed, particularly evident by embryonic stage 40, three days post-surgery.

Initially, our qualitative analysis showed cells with a predominant red signal appeared brightly red, attributed to the higher concentration of red EosFP relative to the un-photoconverted green form. As cell proliferation progressed, and with the ongoing translation of green EosFP, the red EosFP became diluted. (Injected mRNAs can persist and continue to be express during the embryonic stages.) This dilution corresponded to a visible transition in cell coloration, shifting from red to orange. The change in color represented the narrowing ratio of red to green EosFP within the cells. As the division of red-labeled cells continued, this ratio further narrowed, with the red signal becoming increasingly diluted while the green became more prominent. This shift in the red:green EosFP ratio was visually evident as the cell color progressed from orange to yellow and eventually to a light yellow-green hue. After five days, the green EosFP began to dominate, leading to cells appearing as a washed-out green. This final color stage indicated a significant narrowing of the red:green ratio to the point where the green signal predominated. Consequently, this change in the ratio, reflected in the color progression from red to green, rendered the red signal less effective for tracking cell proliferation and the movement of red-labeled cells.

**Figure 7:**
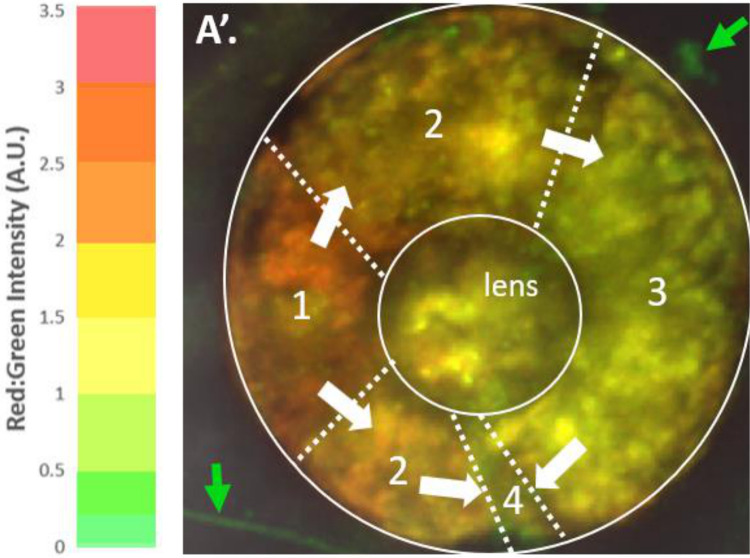
Eos Dilution as an Indicator of Cell Proliferation. Panel **(A’)** illustrates how the red to green intensity ratio can be used to track the dilution of red Eos in the regrowing eye. In panel A’, a st. 46 regrown eye is divided into different regions based on their color and measured red:green EosFP intensity ratio. Red:Green intensity ratios were calculated for each section divided by white dashed lines. Number 1 indicates the initial location of cell growth from the post-surgical RPC niche and is seen as red-orange colored cells. The number 2 indicates the circumferential retinal growth originating from both sides of the initial RPC niche. Cells in this region are orange in color as indicated by their measured red:green intensity ratio. The growth continues to region 3 where the cells take on a yellow and yellow-green hue. Region 4 contains green cells within the optic fissure, indicating the most diluted red Eos within the cells. White arrows indicate the direction of eye growth and decreasing intensity ratio. A colored scale on the left shows a range of intensity ratios and their corresponding colors. Green arrows indicate un-photoconverted green cells and green neural process.

To quantify the relative changes of red Eos to green, we took intensity measurements from a st. 15 photoconverted eye field immediately after photoconversion, measuring both the red and green channel intensities within the eye field. Intensity was measured using Fiji imaging software and expressed in Arbitrary Units (A.U.). To determine the relative intensity ratio of red to green signals from the cells (red:green intensity ratio), we divided the red measurement by the green measurement and recorded the numerical ratio, which corresponded to the color we observed. For the cells within the eye field measured at zero hours post-photoconversion, the values were red = 1194.008 A.U. and green = 341.797 A.U., resulting in a red:green ratio of 3.493, indicative of a pure red hue. To determine the range of colors and their corresponding intensity ratios of red:green Eos observed when examining the combined red and green fluorescent channels, we selected regions of an Eos-labeled st. 46 regrown eye. This eye displayed all colors except the pure red seen at st. 15. We took measurements for all the colors observed when the red and green channels were combined. Our data indicate the following green to red ratios for the colors observed: pure red = 3.5–3, red-orange = 3.0–2.5, orange = 2.5–2, yellow-orange = 2–1.5, yellow = 1.5–1, yellow-green = 1–0.5, and green = 0.5–0 (Figure 7).

**Figure 8:**
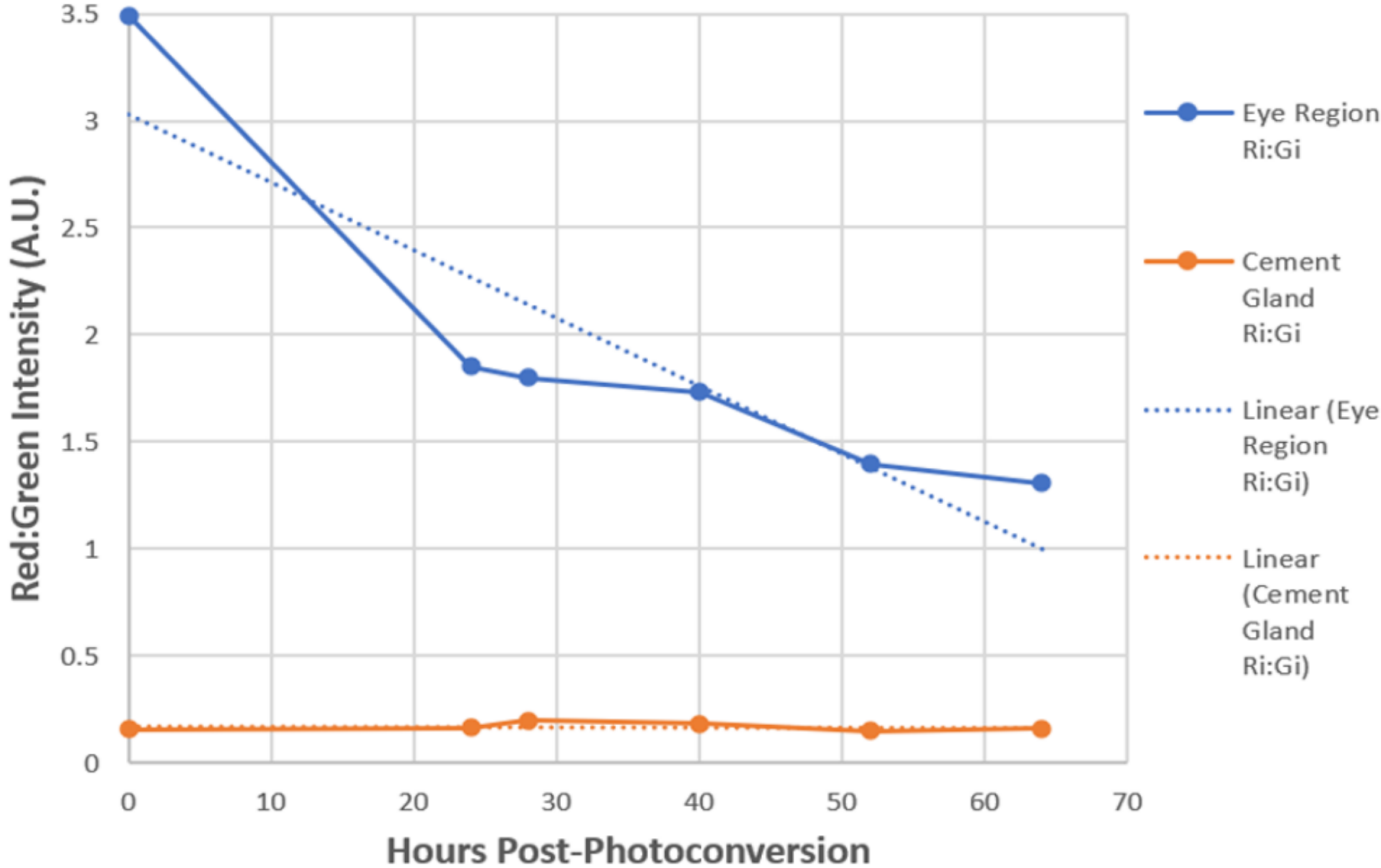
Dilution of Red Eos from Initial Photoconversion Through Eye Regrowth. Figure 8 is a chart that illustrates the dilution of photoconverted Eos over time. At st. 15, the initial photoconversion was made within the eye field. This was seen as a bright red hue within the cells with an intensity of 1194.008 A.U. and a green intensity of 341.797 A.U. The calculated red:green ratio of the eye field at st. 15 was 3.493 A.U., indicating a high red signal to low green signal. Intensity ratios between the red and green channels were calculated within the eye at st. 15, post-surgery, 4 hours post-surgery (hps), 14 hps, 26 hps, 38 hps, and 52 hps. Intensity ratios for the cement gland were calculated and served as an un-photoconverted control for comparison. Both regions and their corresponding intensity ratios were plotted on the scatterplot below (blue line represents the eye, orange line represents cement gland red:green intensity ratios over time). Intensity values on the y-axis correspond to the intensity colors calculated in Figure 6 B. For photoconverted RPCs within the post-surgical wound and regrowing eye, a large decrease in red to green signal is seen within the first 24 hours after photoconversion, with a slower rate of red signal dilution from 24 hours post-photoconversion to 64 hours post-photoconversion. This was likely due to the initial concentrations of EosFP mRNA still being high and actively translating within the first 24 hours to dilute the red signal. The dilution of the red Eos signal after 24 hours can be attributed to cell proliferation with less actively transcribing green Eos to reduce the red:green ratio. The cement gland, however, shows a low red:green ratio through all time points observed, indicating the lack of photoconversion in these cells and their observed bright green color.

Using this scale, we tracked the dilution of the red:green ratio from st. 15 photoconversion to 64 hours post-photoconversion within the regrowing eye (Figure 8). These ratios were taken as an average intensity over the region of photoconverted regrowing eye cells and plotted in Figure 8, represented by the blue line. We observed that 24 hours post-surgery, RPCs within the eye field were red (3.493 A.U.). This dropped to 1.853 A.U. 24 hours post-photoconversion, where the cells displayed an orange-yellow hue immediately after eye removal. At 4 hours post-surgery (28 hours post-photoconversion), the RPCs maintained the orange-yellow hue with an intensity ratio of 1.799 A.U. At 40 hours post-photoconversion, cells of the eye lineage had a red:green ratio of 1.731 A.U.; at 52 hours post-photoconversion, 1.397 A.U.; and at 64 hours post-photoconversion, we observed a red:green ratio of 1.307 A.U., indicating a yellow color. It’s important to note that these were measurements taken as an average over the entire photoconverted region. Some small regions of cells took on ratios in the yellow-green range. This is seen in Figure 7, Panel A’, Region 4, where an equal number of cells displayed a red:green ratio of 0.950 A.U. (yellow-green) and 0.700 A.U. (on the border of pure green). When this is observed, individual cells in that region were measured using the red:green ratio to determine if they were from the photo-converted RPC lineage. An un-photoconverted control region (the cement gland) was measured from st. 15 (time of eye field conversion) to 64 hours after the eye field was converted on the same embryo, serving as a control (Figure 8, orange line). The red:green intensity ratios are as follows, in A.U.: 0.156, 0.163, 0.199, 0.184, 0.187, and 0.160. These values all fall within the lower end of the green range and serve as a way to determine if a cell has been photoconverted or not.

### A Post-Surgical RPC Niche Regrows the Pre-Metamorphic Xenopus Eye

To assess the regenerative capability of the RPC population remaining in the post-surgical wound region, we labeled the right or left eye field red in n=80 st. 15 embryos. Of these, 45 were chosen for eye removal surgeries, selected due to their specific photoconversion within the confines of the eye field. These embryos were photoconverted again at st. 18 in the developing eye to increase the concentration of the red form of Eos before the st. 27 eye removal surgery. Out of these 45 embryos, n=15 survived the entire experimental protocol, which spanned from surgery to imaging at st. 42. The protocol encompassed initial imaging and photoconversion at st. 15, pre-surgical imaging of the eye, post-eye removal imaging, and subsequent analyses at 2, 14, 28, and 36 hours post-surgery (hps). Of these n=15 tadpoles, n=13 exhibited full eye regrowth as per the eye regrowth assay (Kha et al., 2018a), one showed moderate regrowth, and one displayed moderate to weak regrowth. Additionally, a distinct group of 5 tadpoles, also with red-labeled RPCs, were allowed to develop to stage 48, the point of full eye maturity pre-metamorphosis. Imaging of these tadpoles was conducted less frequently to preserve the integrity of the red label. Within this group, one tadpole experienced weak regeneration, while four exhibited full to moderate regrowth. In cases of eyes categorized as fully, moderately, or weakly regrown, all exhibited regeneration wherein more than 80% of the eye could be distinctly traced to the red-labeled retinal cell lineage from before the surgical removal of the eye and after surgery, as well as at 2 hours post-surgery (2 hps) and every 12 hours until stage 42, is described below.

### St. 27 Pre-Surgery

**Figure 9:**
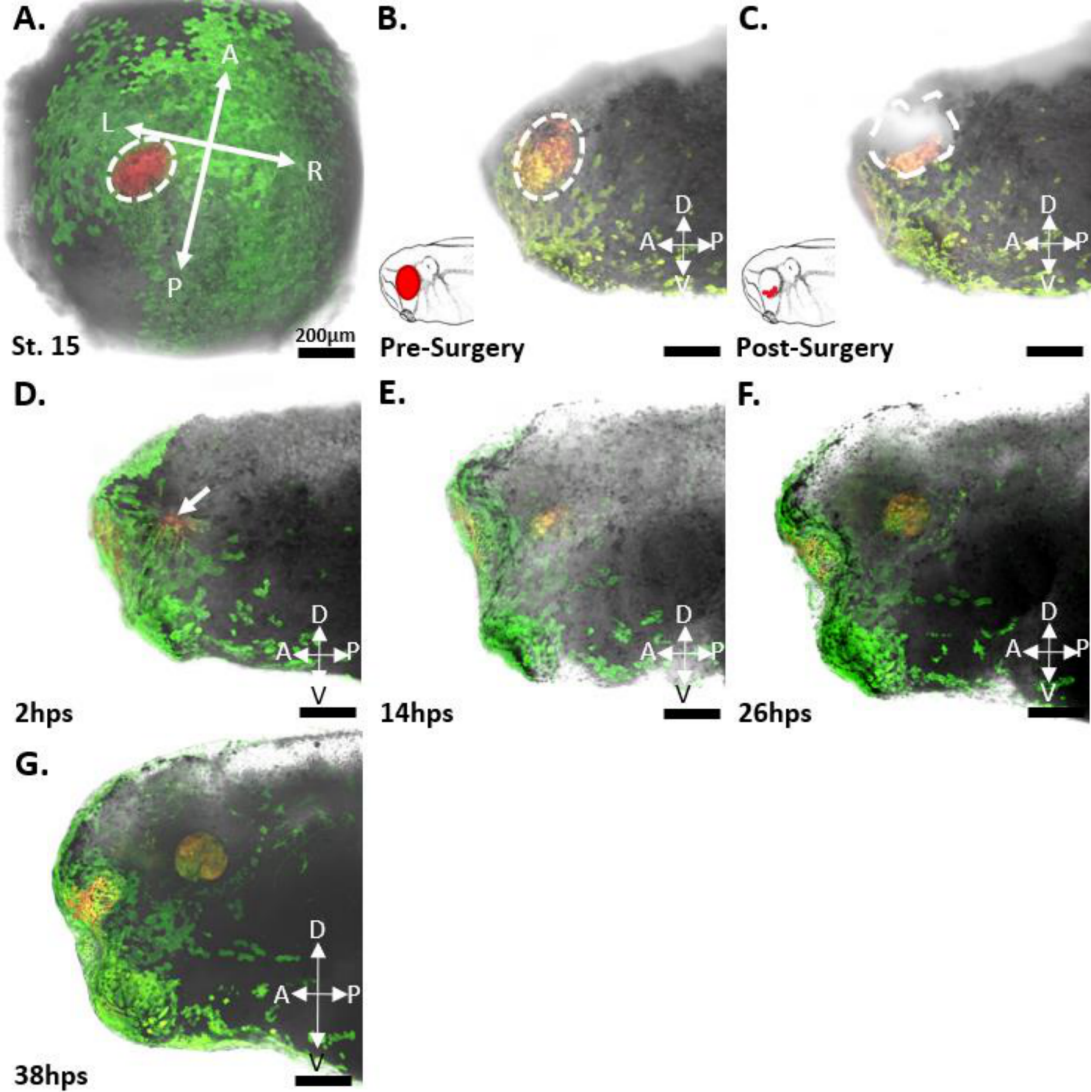

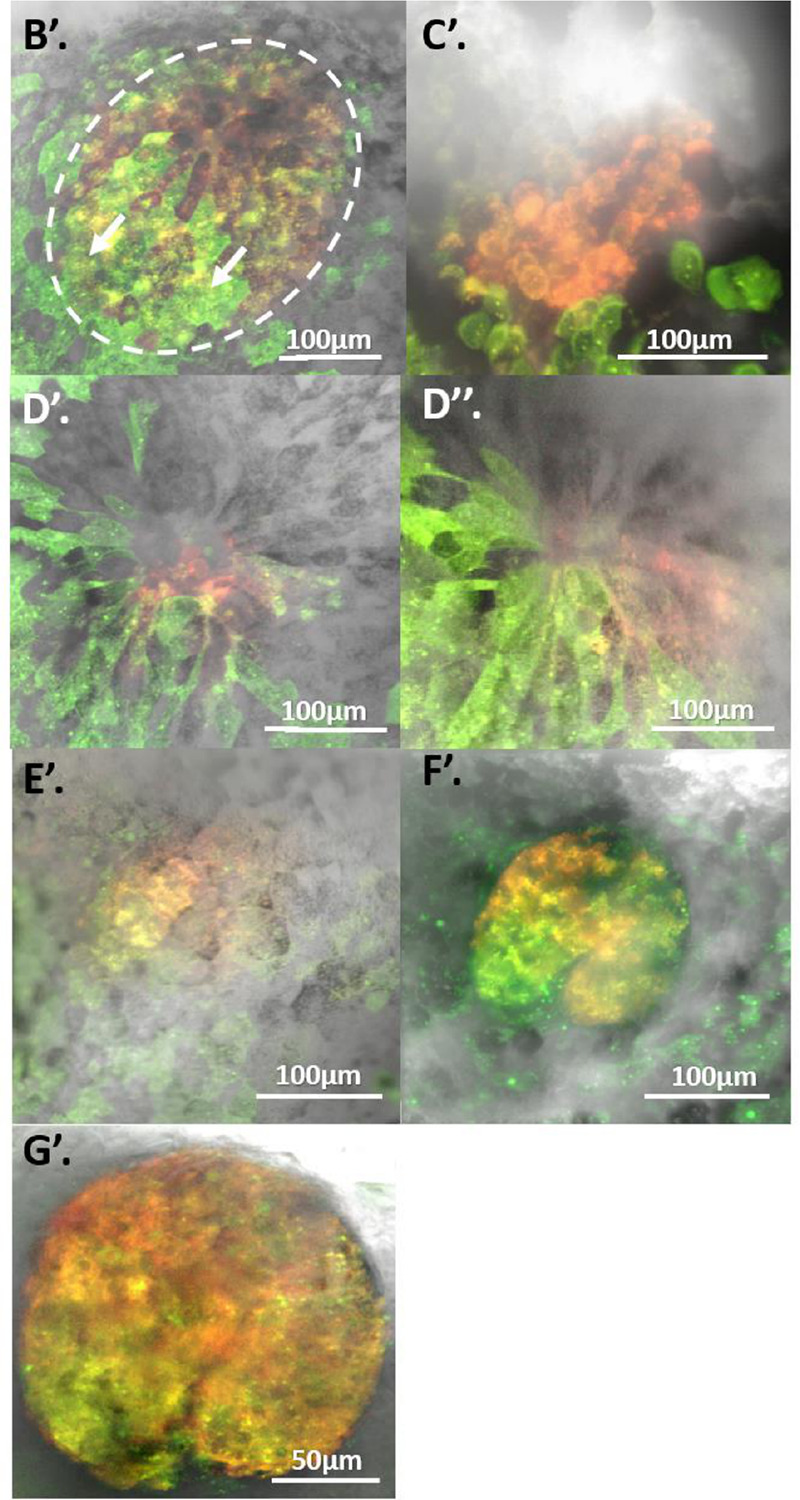
A Post-Surgical Population of Retinal Progenitor Cells is the Primary Source for Pre-Metamorphic Tadpole Eye Regrowth. **(A)** st. 15 Xenopus embryo expressing the green form of EosFP within the cells of the neural plate and non-neural ectoderm. The green fluorescent protein was permanently photoconverted from green to red with UV light in the dashed oval region, indicating the location of the left eye field. Arrowed lines show orientation of the anterior–posterior axis (A<->P axis) and Right -Left side of the embryo (L<->R). **(B)** Panel B illustrates the same embryo now developed a day later, at embryonic stage 27 prior to an eye removal surgery. The red labeled retinal progenitor cells (RPCs) are seen within optic vesicle (dashed oval line), while green-labeled non-retinal cells are seen surrounding the eye and migrating on the surface of the optic vesicle. The drawn insert of the Xenopus tailbud embryo diagrams the location of red RPCs within the developing eye. **(C)** Panel C displays the same live embryo following an eye removal surgery. The dashed oval surrounds the post-surgical wound, which contains a small population of bright red-orange RPCs in the ventral portion of the wound. Green cells of the epidermis are found surrounding the wound region on the anterior and ventral side. The drawn insert shows the location of the RPCs within the wound. **(D)** The labeled tadpole was imaged again 2 hours post-surgery (hps). The surrounding epidermis (comprising green and non-labeled cells) has taken on an elongated morphology and migrated to cover approximately 75% of the wound. Red-labeled RPCs are found underneath the encroaching wound epidermis and in a central circular region not yet covered by the epidermis. The single white arrow indicates the location of red RPCs. **(E)** The tadpole was imaged again at 14 hps. A protrusion from the surface of the embryo, comprising red, orange, and yellow portions of the regrowing eye, is now visible. The red group of RPCs is flanked on either side by cells with a reduced concentration of the red form of EosFP, appearing yellow-orange anteriorly and orange posteriorly. The red, orange, and yellow region of regrowth forms a crescent shape, with the opening facing the antero-ventral region of the tadpole. Green and non-labeled epidermis cells have covered the wound region and now superficially cover the regenerating eye. The central portion of the crescent contains a faintly green-labeled cell (indicated by the green arrow) surrounded by orange and red RPCs (indicated by the orange arrow). The white dotted line demarcates the crescent-shaped area of eye regrowth. **(F)** At 26 hps, the regenerating eye assumes a round shape. The crescent’s opening has nearly closed, with yellow and yellow-green cells on the antero-ventral portion of the eye. The ventral part of the eye (slightly posterior) consists of orange-yellow cells. This part of the regenerating eye resembles the optic fissure seen in early stages of eye development but has only started to close in the eye’s inner ring, nearest the presumptive lens. The eye’s central portion is now filled with orange-labeled cells, with a developing border evident from surrounding eye progenitors. Green and non-labeled cells have migrated further across the eye and beyond to the posterior part of the tadpole. **(G)** The same tadpole imaged at 38 hps shows a regenerating eye with an even more rounded morphology. Red cells in the dorsal portion of the eye have divided, diluting their concentration of the photoconverted red Eos, visible as dark, orange-colored cells in the eye’s dorsal region. This area of initial regrowth is flanked by lighter shades of orange and yellow cells. The ventral part of the eye is covered by green cells (as seen in the z-stack of the image), with dilute yellow-green cells having proliferated to close the optic fissure. The central portion of the eye contains orange cells, and a distinct round lens is starting to form. Green cells have spread further over the surface of the eye and along the tadpole’s left side. Orange and light green cells within the eye’s central portion have formed a round, regenerating lens. The black lines in the bottom right corner of the panels indicate 200 μm scale bars. D= dorsal, V= ventral, A= anterior, P= posterior anatomical orientations. Drawn images are modified from Nieuwkoop & Faber (1994) Normal Table of Xenopus laevis. Drawings modified from Nieuwkoop and Faber (1994). Panels B’-G’ are expanded views of the eye region in panels B-G. Green arrow in B’ marks the location where many green cells have migrated to cover the surface of the optic vesicle. Panel D” is the fully closed wound at 5 hps. D= dorsal, V= ventral, A= anterior, P= posterior anatomical orientations. Drawn images are modified from Nieuwkoop & Faber (1994) Normal Table of Xenopus laevis. Drawings modified from Nieuwkoop and Faber (1994).

**Figure 10:**
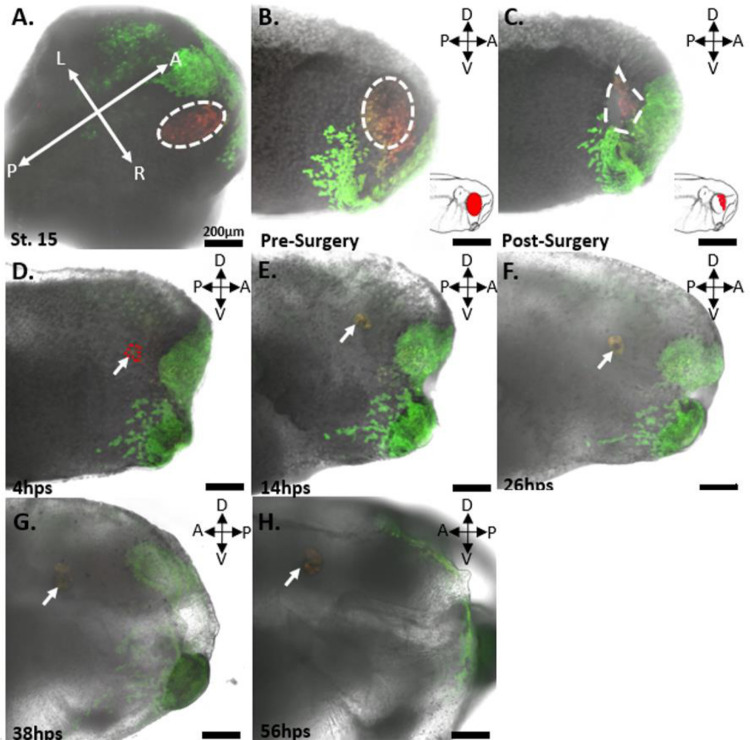

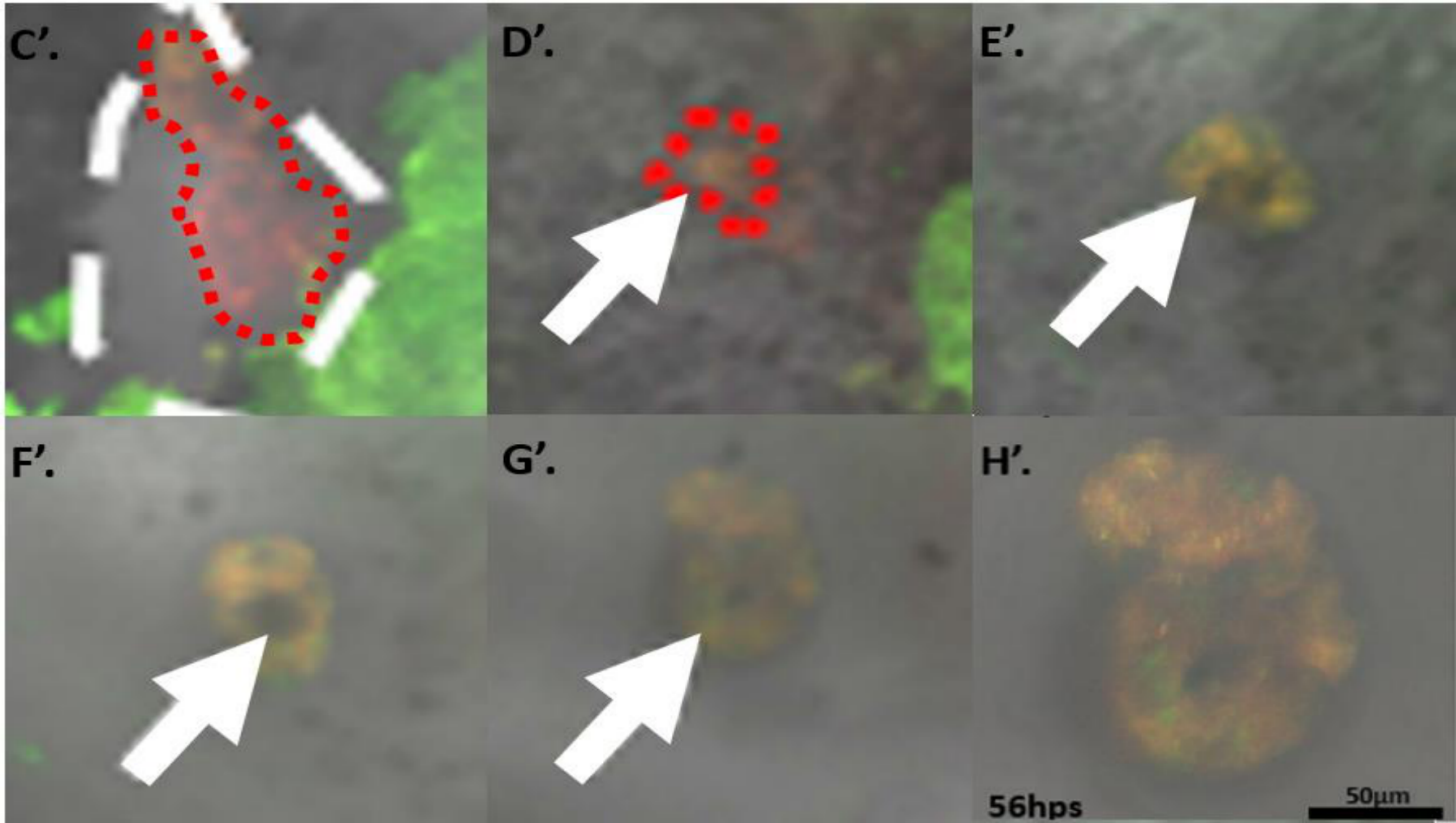
Weak Eye Regrowth is Subserved by a Post-Surgical RPC Population. **(A)** An image of a stage 19 Xenopus embryo expressing the green form of EosFP within the cells of the developing nervous system. The green fluorescent protein was permanently photoconverted from green to red with UV light in the dashed oval region, indicating the location of the right developing eye. Arrowed lines show the orientation of the anterior–posterior axis (A<->P axis) and the right-left side of the embryo (L<->R). **(B)** Panel B illustrates the same embryo one day later, at embryonic stage 27. The red-labeled retinal progenitor cells (RPCs) are visible within the right optic vesicle (dashed oval line). Green-labeled non-retinal cells are found surrounding the ventral-posterior part of the eye, within the cement gland, and olfactory region. The drawn insert of the Xenopus tailbud embryo diagrams the location of red RPCs within the developing eye. **(C)** Panel C displays the same live embryo after an eye removal surgery was performed. The dashed line encircles the post-surgical wound, which contains a small population of red RPCs in the anterior periphery of the wound. Green epidermal cells are found surrounding the wound on the ventral side and bordering the anterior wound from the presumptive olfactory placode. The drawn insert diagrammatically shows the location of RPCs within the wound. **(D)** The labeled embryo was imaged again 4 hours post-surgery (hps). The surrounding epidermis (non-fluorescent/ lightly pigmented cells) were found to cover 100% of the wound. A cluster of 5 red-labeled RPCs (50 μm total diameter) is found beneath the now-healed epidermis. A faint red signal from the other remaining RPCs was visible but occluded by the overlying tissue. The white arrow and red dashed line indicate the location of red RPCs. **(E)** The embryo was imaged again at 14 hps. An orange 90 μm crescent-shaped group of cells with a non-labeled center is visible from the embryo’s surface. The opened end of the crescent is oriented ventrally. **(F)** At 26 hps, the regenerating eye fragment assumes a more circular shape. The opening of the crescent has now closed with light orange-labeled cells on the ventral portion of the eye, while the eye fragment has grown to 120 μm at its widest point. **(G)** The same embryo, imaged at 38 hps, shows an eye with a round morphology on the ventral (bottom) 3/4 of the eye. The dorsal (top) part of the eye has expanded, making the regenerating eye fragment 130 μm at its widest point. No cells are seen populating the center of the eye fragment, even though the fissure at the ventral portion of the eye has closed. **(H)** At 56 hps, the orange eye fragment has grown to 150 μm, with some thickening on the dorsal portion of the eye. The ventral portion of the eye fragment has become more circular but remained the same diameter as at the 38 hps time point. No lens is evident. Panels **(C’-G’)** are cropped images of the regrowing eye region found in panels C-E. C’-G’ share the same field of view. **(H’)** An image of the eye fragment with a 50 μm scale bar for size reference taken with a 20x objective. Orange cells are found throughout the eye fragment, with some light green interspersed cells. No lens is present, and the fragment is misshapen, indicating weak regeneration. Black lines in the bottom right corner of the panels indicate 200 μm scale bars, unless otherwise noted. D= dorsal, V= ventral, A= anterior, P= posterior anatomical orientations. Drawn images were modified from Nieuwkoop & Faber (1994) Normal Table of Xenopus laevis. Drawings modified from Nieuwkoop and Faber (1994).

**Figure 11:**
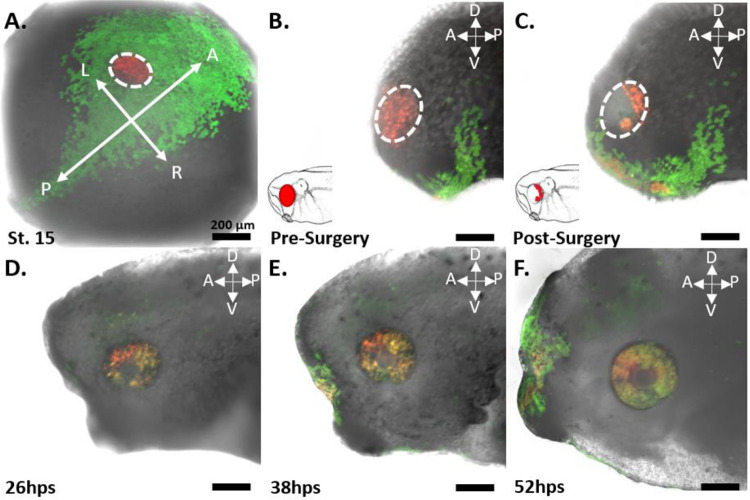
A Remaining Population of Post-Surgical RPCs Aids in Full Eye Regrowth. **(A)** Stage 15 Xenopus embryo expressing the green form of EosFP within the cells of the neural plate and non-neural ectoderm. The green fluorescent protein was permanently photoconverted from green to red with UV light within the dashed oval region, indicating the location of the left eye field. Arrowed lines show the orientation of the anterior–posterior axis (A<->P axis) and right-left side of the embryo (L<->R). **(B)** Panel B illustrates the same embryo, developed a day later at embryonic st. 27. The red-labeled retinal progenitor cells (RPCs) are seen within the left optic vesicle (dashed oval line). Green-labeled non-retinal cells are observed in the cement gland and on a small strip extending from the cement gland along the left side of the embryo. The inserted drawing of a Xenopus tailbud embryo indicates the location of the red RPCs within the developing eye. **(C)** Panel C shows the same live embryo after an eye removal surgery. The dashed oval surrounds the post-surgical wound with a small population of red RPCs remaining within the posterior portion of the wound, beneath the level of the surrounding epidermis. Green epidermal cells are seen migrating towards the wound from the anterior side. The inserted image shows the location of red RPCs within the wound. **(D)** The labeled tadpole was imaged again at 26 hps. The surrounding green-labeled epidermal cells are faint and barely visible on the left side of the embryo. A region of red-labeled RPCs, approximately 200 μm across, is found in the antero-dorsal quarter of the regrowing eye. This red region is flanked on either side by orange and then yellow cells, forming an almost closed crescent. An optic fissure is observed on the ventral portion of the regrowing eye, with yellow RPCs not yet filling the fissure to close the eye. **(E)** The embryo was imaged again at 38 hps. The red RPC region has shrunk to approximately 125 μm, while the orange and yellow cells of the regrowing eye have expanded the crescent shape ventrally to form a nearly circular, enclosed eye. The ventral portion of the optic fissure is not yet fully closed, with red-orange-colored RPCs seen within the regrowing lens region. **(F)** At 52 hours post-surgery the regenerating eye has adopted a round shape. The once-red antero-dorsal quarter of the eye has now diluted the red label to an orange color, approximately 100 μm across. This orange area is flanked by yellow and light green/yellow cells near the closed optic fissure. The lens is also present and composed of orange and yellow cells on the periphery. Black lines in the bottom right corner of the panels indicate 200 μm scale bars. D= dorsal, V= ventral, A= anterior, P= posterior anatomical orientations. Drawing modified from Nieuwkoop and Faber (1994).

In the st. 27 embryos, the red-labeled eyes predominantly contained red-labeled cells on the surface before eye removal surgery (Figure 9 and Figure 10 Panel B; Figure 11, Panel B). Notably, in n=5 cases, green cells were observed migrating onto the surface of the eye from st. 18 to st. 27, presumably to aid in lens formation (Figure 9, Panel B and B’). This migration was especially pronounced in n=4 tadpoles that did not undergo a second round of photoconversion at st. 18. Nonetheless, at st. 27 the optic vesicles predominantly labeled red, and the overlying green cells were removed during the surgical procedure, rendering them inconsequential. In n=3 embryos, red-labeled cells were also detected within the olfactory placode, likely due to their proximity to the eye field at st. 15 (Figure 9). These cells were monitored throughout the imaging series; however, they did not appear to integrate into the regrowing eye. The possibility for cells to migrate into the regrowing eye during our imaging timepoints may have occurred, however we did not see this reflected in the internal anatomy of the regenerated eye. Interestingly, in one case, clonally related cells originating from the olfactory region formed a path directly into the wound area of n=1 tadpole. Unfortunately, this tadpole perished 2 hours post-surgery, precluding any conclusion about potential contributions to eye regrowth.

The patterns of the green Eos labeled cells varied slightly from one tadpole to another. All embryos had green labeled cells forming the neural plate, the presumptive olfactory region, the cement gland, and varying amounts of green cells spreading across the surface ectoderm. In all experimental embryos, the green form of the protein was photoconverted to the red form within the eye. The green form of Eos was then found within the brain, the olfactory bulb, the cement gland, and in some instances, in the epidermis surrounding the protruding optic vesicle and eye at later stages of development.

### St. 27 Post-Surgery

After a st. 27 eye removal surgery, the 15 tadpoles were immediately imaged again to determine the location of the remnant RPC niche (Figure 9 and Figure 10, Panel C; Figure 11, Panel C). Red-labeled progenitor cells were found only within the wound region of the removed optic vesicle and within the extirpated eye. The distribution of red cells within the wound varied from tadpole to tadpole, but in each of the n=15 tadpoles, a variable amount of red-labeled tissue was found to circumscribe the interior of the oval wound (Figure 9 and Figure 10, Panel C; Figure 11, Panel C). The red-labeled cells extended from the level of the surgical incision in a crescent shape and down to a depth of no more than 200 μm (Figure 4). The retinal progenitor cells partially encircled the opening of the optic stalk, with some red cells surrounding but not entering the opening of the stalk at the 200 μm depth. For n=14, the crescent-shaped population of post-surgical retinal progenitor cells (RPCs) came into contact with the ventral portion of the eye (Figure 9 and Figure 10, Panel C; Figure 11, Panel C), while n=1 had a cluster of red-labeled cells within the dorsal-posterior region of the eye.

### 2 Hours Post Surgery

A typical wound healing process in an eye that fully regrows is observed at 2 hours post-surgery (hps) as depicted in Figure 9, Panel D. At 2 hps, both green and non-labeled cells surrounding the wound have changed morphology, elongated, and migrated to cover approximately 75% of the wound. Red-labeled RPCs are found underneath the encroaching wound epidermis and in a central circular region not yet covered by the epidermis. Contrasting with the elongated cells of the closing wound epidermis, the red RPCs retain a round shape and do not appear to migrate from their position in the wound. However, small movements of red RPCs are evident during a lengthy (5-minute) imaging session at 2 hps. This was observed for all n=15 tadpoles and seems to coincide with the direction of the overlying epidermis towards the central circular region of the wound. This did not significantly alter the shape of the post-surgical RPC population but compacted it by a few cell diameters.

For n=2 tadpoles, an additional image was generated 6 hours post-surgery. At this point the wound had completely healed, and the surrounding epidermis had resumed a normal morphology. Cells overlaying the wound reverted from the elongated shape, previously aligned radially with the central portion of the wound, to a normal round shape. The epidermal cells completely covered the wound, with the red progenitor cells faintly visible beneath. These red cells remained within the same general footprint of the original colony of post-surgical retinal stem cells; however, new growth was observed. This growth is evidenced by an expansion of orange cells from the red retinal progenitor cell (RPC) region. The orange color indicates that the cells have divided, thereby diluting the amount of red Eos in relation to green Eos within the cell. This growth was minimal, with only a few cells on the periphery of the red RPC colony being observed. This observation of new cell growth was more evident by 14 hps. While lateral growth was minimal at this point, the thickness of the eye in the z-axis had grown from being flush or below the top of the wound to protruding out slightly by 40 μm above the surface of the surrounding skin from 2 hps.

### 14 Hours Post Surgery

At 14 hps, increased growth from the red retinal progenitor cell colony was observed (Figure 9 and Figure 10, Panel E). All regrowing eyes, including the weakly regenerated one, adopted a crescent-like shape. Although the size of this crescent-shaped regrowth and the width of the optic fissure varied based on the quantity of remaining red RPCs post-surgery, the optic fissure (unclosed portion of the regrowing eye) consistently faced ventrally in all 15 experimental tadpoles, regardless of where the cluster of post-surgical RPCs were found in the wound. This suggests that the surgical procedure may not influence the general pattern of regrowth or the ventral orientation of the optic fissure that was observed. Notably, new growth was observed as orange cells near the opening of the crescent, flanking a central group of red cells. The central cells within this ‘C’ shape expanded, contributing to additional height, while the peripheral areas of the RPC colony exhibited outward expansion. This expansion was detectable by a change in the red lineage tracer color, shifting from a dark reddish-orange to a light orange, signifying the dilution of the tracer by new cell growth.

### 26 Hours Post Surgery

By 26 hours post-surgery (26 hps), the observed expansion pattern had continued, with further thickening of the central more red colored portion of the colony. The ends of the previous crescent shape were now clearly oriented towards the tadpole’s ventral portion. Additional growth was noticeable at both ends of the previously crescent-shaped colony, with the cells of the new ventral growth displaying a lighter orange or yellow color compared to the 14 hps growth (Figure 9 and Figure 10, Panel F; Figure 11 Panel D). This new growth may be similar to the ciliary marginal zone seen during development which contains the most proliferatively active growth of the expanding eye.

### 38 Hours Post Surgery

By 38 hps, the crescent expansion pattern had continued, with further thickening of the central more red-colored portion of the colony (now orange). The ends of the previous crescent shape were now clearly forming the optic fissure of the eye. Additional growth was noticeable at both ends of the previously crescent-shaped colony, with the cells of the new ventral growth displaying a lighter orange or yellow color compared to the 14 hps growth (Figure 9 and Figure 10 Panel G; Figure 11 Panel E). This new growth may be similar to the ciliary marginal zone seen during development which contains the most proliferatively active growth of the expanding eye.

### 72 Hours Post Surgery (Multiphoton Analysis)

By 72 hours post-surgery in multiphoton imaged tadpoles, the ventral portion of the eye had fully closed to form a ring, due to the addition of newly proliferated retinal cells presenting a yellow to light green color. Presumably, this region represented the regenerated optic fissure, and was the thinnest portion of the regrown eye, with all other parts showing increased cell density. The eye at this stage of regrowth measured a total depth of 250 μm, from the most lateral to the most medial portion.

**Figure 12:**
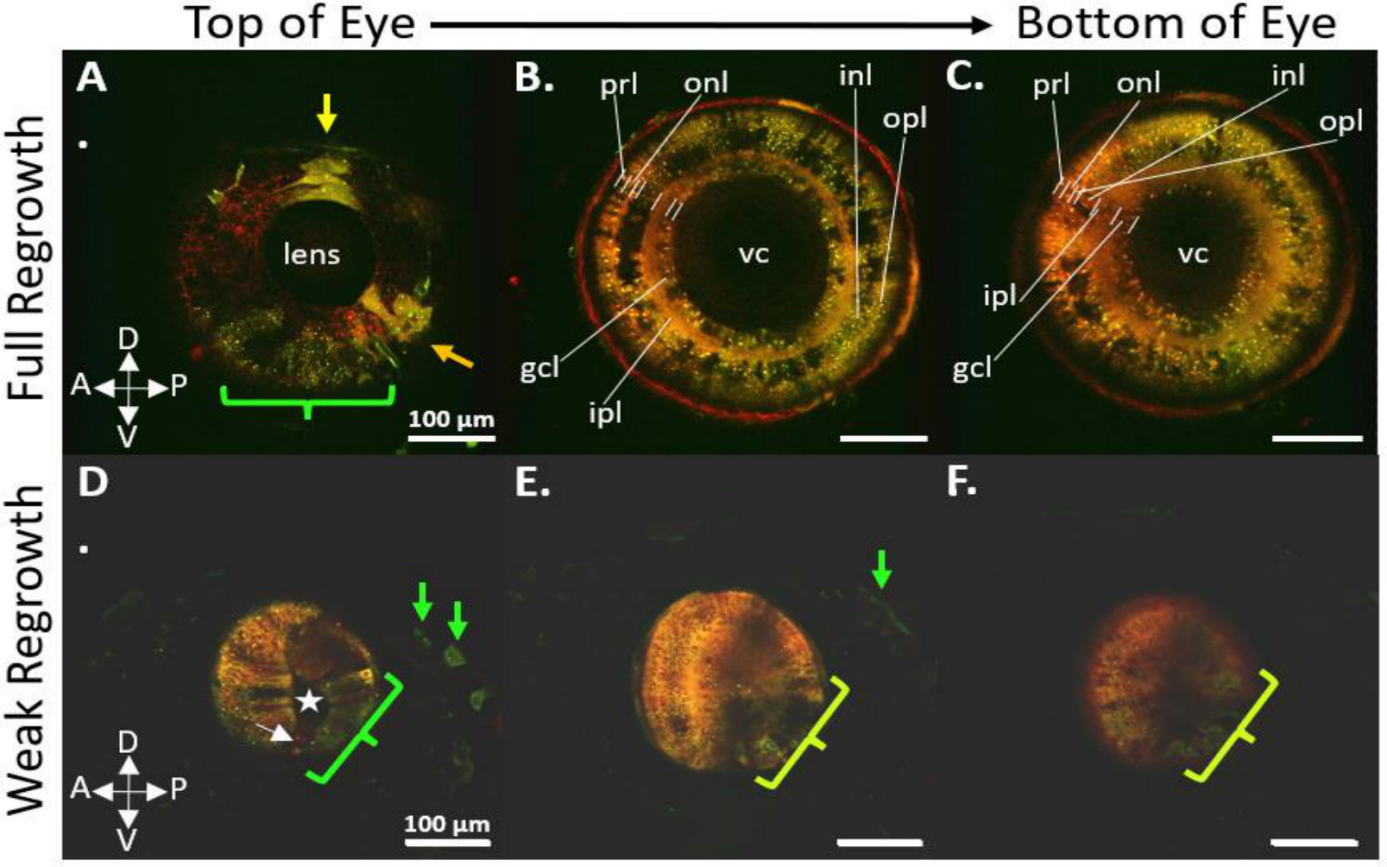
Post-Surgical RPCs Fate Mature Cells of the Eye. **(A-C)** Optical sections taken with a multiphoton microscope through the depth of a regrown tadpole eye, categorized as fully regrown, at 72 hours post-surgery. **(A)** Panel A depicts the most superficial layer of the eye. The first optical section was taken at the level of the lens furthest from the brain, lateral to the tadpole’s midline. Red labeling is found throughout the superficial layer of the eye. Larger orange and yellow-green labeled cells are seen in the dorsal and ventral portions of the eye (indicated by yellow and orange arrows). Green labeled cells are present in the most ventral portion of the eye (green bracket). No optic fissure is observed in the regenerating eye at 6 hps. **(B)** Panel B reveals the internal anatomy of the eye in both red and green channels, 50 μm deeper than panel A. A gradient of red label from red to lighter shades of orange and yellow/green is seen across the regrowing eye. The mature retinal anatomy is present and is depicted by the different retinal layers, including the photoreceptor layer (prl), outer nuclear layer (onl), outer plexiform layer (opl), inner nuclear layer (inl), inner plexiform layer (ipl), and ganglion cell layer (gcl). This optical section was taken at a depth 50 μm below panel A. Some cells in the onl are not visible due to the shallow depth of the section within the eye and the contoured anatomy of the retina. **(C)** A gradient of red label from red to lighter shades of orange and yellow/green is observed across the regrowing eye at a 150 μm depth. This gradient, seen in panels A-C, indicates that all mature retinal cell layers have originated from RPCs left over at the time of eye removal. **(D-E)** Panels D-E depict optical sections of a weakly regenerated eye at 72 hps. **(D)** Show the regenerate eye at its most superficial level. No lens is present in the central portion of the eye (white star). The eye has not fully closed as evidenced by an optic fissure (white arrow) and the eye is slightly misshapen. Green arrows point to green cells outside the retinal lineage. **(E)** The same eye was imaged 50 μm lower. The typical retinal layering, as seen in panels A-C, is partially evident but not fully developed. The optic fissure at this depth still has not closed. Green-yellow brackets indicate areas of diluted red Eos lineage tracer. A green arrow indicates non-photoconverted epidermal cells. **(F)** Panel F depicts the same weakly regenerated eye 100 μm down from the surface of the tadpole. Approximately ¾ of the eye at this depth are labeled red to orange, with ¼ of the eye presenting a green signal greater than a red signal. Yellow-green regions are indicated by yellow-green brackets. White bars indicate 100 μm scale bars for all images. D= dorsal, V= ventral, A= anterior, P= posterior orientations of the regrowing eye.

Among the five tadpoles (n=5) that regenerated their eyes and survived until 72 hps for multiphoton analysis, the regenerated eyes exhibited the morphology of a completely mature tadpole eye. On average, the regrown eyes were 80% the size of the contralateral unoperated control eye, featuring a normal cornea, lens, closed round morphology, light orange-yellow labeled optic nerve, and attached extraocular muscles. Live imaging revealed the regrown eye executing constant eye saccades before the complete onset of tricaine anesthesia (Figure 14), suggesting innervation and functionality of the extraocular muscles. The mature morphology of the regenerated eye was further corroborated through optical sectioning of the 72 hps stage 48 tadpoles (Figure 12). Fully regenerated eyes (n=5) and weakly regenerated eyes (n=3) were imaged from the lateral-most part of the eye, starting at the superficial layer of the lens and continuing through the full depth of the eye towards the brain in 5 μm increments. The resulting optical sections from the fully regenerated eye exhibited the typical retinal morphology found in an unoperated eye, with all retinal layers, including the ganglion cell layer, inner plexiform layer, inner nuclear layer, outer plexiform layer, and photoreceptor layer, clearly present. Cells within each layer and throughout the depth of the eye appeared light orange, with the ventral portion of the eye containing cells ranging from a lighter yellow to green. No visible sign of an optic fissure was found in the ventral region of the eye, indicating complete closure. Nevertheless, the ventral portion of the eye contained the largest number of yellow-green or green-only cells at stage 48.

For the weakly regenerated eye, the results were similar, but the optic fissure remained open, a lens had not developed, and the retinal layers, while present, were less developed than in the fully regenerated eye (Figure 12, Panel E). While the outermost layer of the weakly regenerated eye did contain cells resembling a photoreceptor morphology, an outer plexiform layer, an inner nuclear layer, an inner plexiform layer, and possibly a ganglion cell layer, these layers were less defined than in the fully regenerated eye. Additionally, the cells within each layer (except the photoreceptor layer) were ambiguous in their morphology.

### RPC Niche Impact on Moderate and Weak Eye Regrowth Following Surgery

In the category of moderately regrown eyes, including those bordering on being classified as fully regrown, a consistent pattern of eye regrowth was observed, albeit at a delayed rate compared to fully regrown counterparts. At 14 hours post-surgery (14 hps), red-labeled cells remained within the wound region, exhibiting minimal growth or color change. However, by 26 hours post-surgery (26 hps), these cells began to form a crescent-shaped pattern similar to that observed in fully regrown eyes (Figure 9, Panels E’-G’). This pattern was smaller in diameter but followed the same gradation of color from red to orange, then yellow, and finally green at the youngest and most proliferative part of the eye.

These moderately regrown eyes were less than 80% the size of the contralateral, unoperated eye but displayed all the internal retinal cell types, organized in the stereotypical retinal anatomy. Notably, 2 of these moderately regrown eyes lacked a lens, but all exhibited red cells contributing significantly to the majority of the eye’s regrowth.

In cases categorized as weakly regrown, a crescent-shaped morphology was possibly discernible in n=2 of the eyes (Figure 10, Panels E’-F’). However, these eyes did not exhibit the normal layered retinal anatomy; it was partially evident but not fully developed (Figure 12, Panels D-F), as seen in more fully developed eyes (Figure 12, Panels A-C). In all instances of weakly regrown eyes, the cells within these structures were primarily labeled red, with a scattering of interspersed green cells.

### Additional Cell Populations May Contribute to Xenopus Embryonic Eye Regrowth

Although our findings demonstrated that the majority of the *Xenopus* embryonic eye regrew through the utilization of a preexisting retinal progenitor cell niche in the existing eye location, the rapid proliferation and dilution of cells expressing Eos lineage tracer might be masking the involvement of other cell populations in eye regrowth. Initially, we attributed the green coloration in the ventral portion of the eye at 38 and 72 hours post-surgery (hps) to the dilution of red Eos and the accumulated post-photoconversion translation of green EosFP (Figure 9, Panel G’; Figure 12, Panel A). Additionally, we observed that the regrown lens contained both red and green labeled cells.

To ascertain whether the regrown lens originated from the surrounding neural epidermis, akin to normal development, or if the RPC niche contributed to lens regeneration, we photoconverted cells surrounding the st. 27 eye while leaving the eye green. We then removed the green eye to determine if red-labeled epidermal cells aided in lens regrowth. This process was conducted with n=2 embryos, which were monitored at 2 hps, 18 hps, and at st. 42 then subsequently examined under the multiphoton microscope.

**Figure 13:**
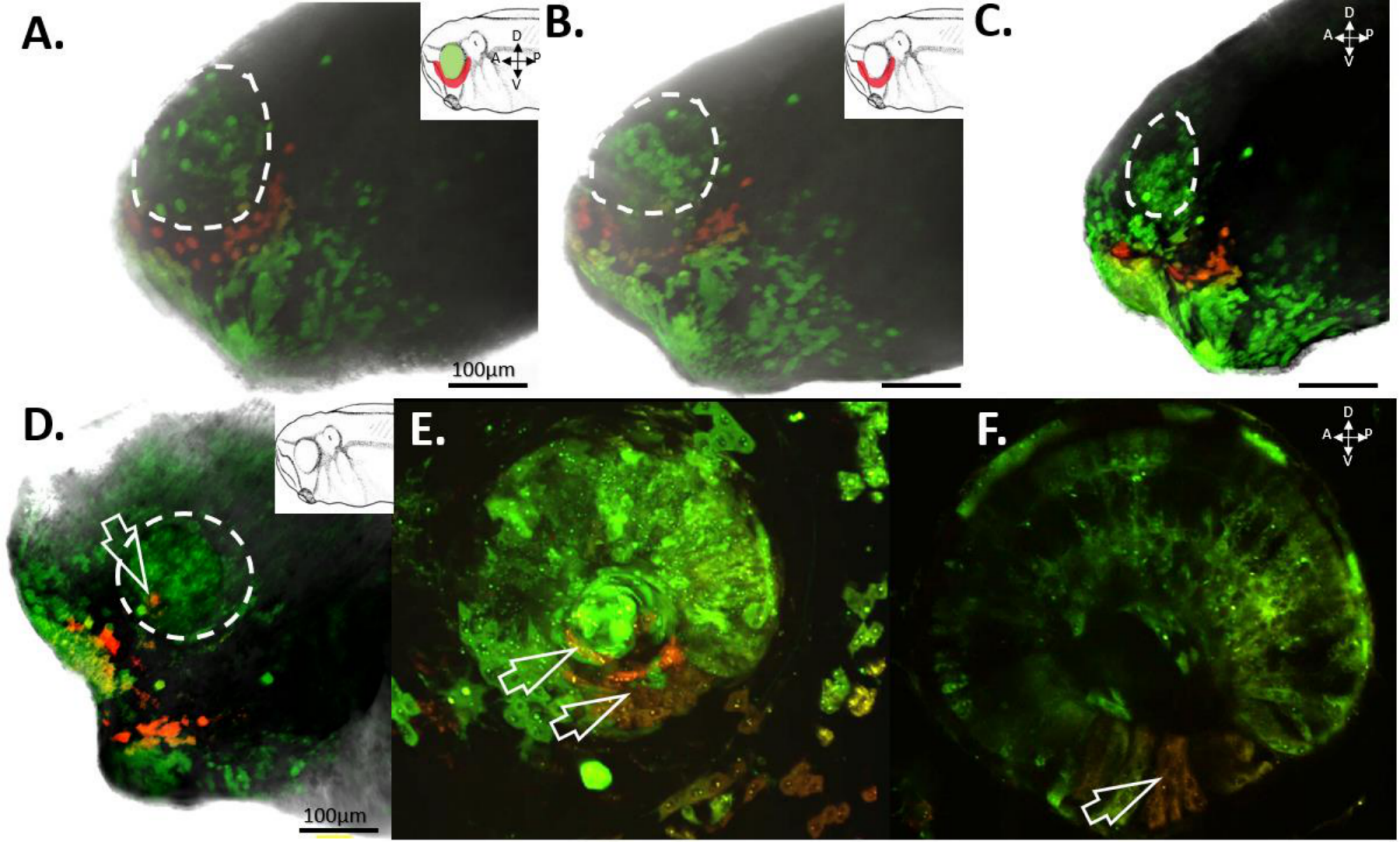
Additional Cell Populations May Contribute to Xenopus Pre-Metamorphic Eye Regrowth. Panel **(A)** Shows a st. 27 tadpole with a green labeled eye and red labeled epidermal cells surrounding the ventral half of the eye. **(B)** An eye removal surgery was performed and green cells are seen within the wound region while red epidermal cells are outside the wound. **(C)** The same tadpole 2 hps shows the red epidermal cells remain outside the wound. **(D)** The same tadpole at 18 hps shows a circular green eye starting to regrow and one red labeled cell on the surface of the eye. Arrowhead indicates location of red cell. Panel **(E)** is a multiphoton image of the regrown eye at st. 42. The eye has regrown with green RPCs but has red cells in the lens and superficial surface of the eye. Panel **(F)** is the same eye at st. 42 halfway down the depth of the eye. Arrowhead points to red-labeled cells integrated into the anatomy of the eye. The region of integration is the ventral region of the eye in the optic fissure. Annotations: D= dorsal, V= ventral, P= posterior, A= anterior, dashed circle= eye in panel A, wound in panel B, regrowing eye in panels C and D. Insert diagrams show orientation of tadpole and location of red labeling in relation to the green eye. Drawing modified from Nieuwkoop and Faber (1994).

In these embryos (n=2), we found that the lens regrew, incorporating some of the epidermal cells, as anticipated (Figure 13, Panel E). Unexpectedly, red-labeled cells were also observed incorporating into the ventral portion of the retinal anatomy (Figure 13, Panel F). This incorporation was noted in the ventral region of the closing optic fissure, the same area where we had previously observed green cells during our RPC eye regrowth assay. Although our sample size is limited to n=2, these findings suggest intriguing implications for *Xenopus* eye regrowth. If substantiated, these findings would imply that st. 27 tadpole eye regrowth may involve non-RPC populations. It may also include a second mode of regeneration like transdifferentiation, where cells from outside the retinal lineage play a role in the regrowth process.

## Discussion

Despite previous research identifying the steps involved in embryonic eye regrowth, a definitive source for retinal progenitor cells (RPCs) has remained elusive until now (Kha et al., 2018a; Kha et al., 2018b; Kha et al., 2019; Kha et al., 2020; Kha et al., 2023). Our observations revealed that a small population of post-surgical RPCs remaining after an ablative eye surgery can regenerate an age-appropriate eye within the previously reported 3–5 day period, supporting our initial hypothesis. Importantly, our study offers a substantial extension to previous work by Kha et al. (2018a) and Kha et al. (2020) by visualizing the location of post-surgical eye progenitors *in vivo* and through the processes of regeneration.

In vivo imaging allowed us to locate RPCs within each individual tadpole’s wound, an observation not achievable with previous methods. Benefiting from live imaging, we observed that, although the surgical cuts for all tadpoles were made in the same orientation (starting in a ventral to anterior direction), the spontaneous lift of the optic vesicle from the wound—a process where the optic vesicle becomes unattached and moves away from the wound site on its own—exhibited a stochastic nature. This variability in the detachment and subsequent removal of tissue within the wound occurred without any external intervention in some instances, indicating that the lifting of the optic vesicle has a random and unpredictable component that can vary from one tadpole to another. As expected, this resulted in varying amounts of RPCs on either the anterior or posterior side of the eye. These progenitor cells typically reside within the ventral portion of the wound and extend along the anterior or posterior contour of the oval wound, forming a crescent shape. This outcome may be attributed to consistency in the surgical procedure specific to one individual and may vary from person to person. Interestingly, our data suggest that the anterior or posterior location of the RPCs did not significantly impact the overall size of the regrown eye. Instead, the total number of RPCs remaining within the area of the wound post-surgery appeared to be a more influential factor in eye regrowth, where the average RPC cell volume remaining within the wound immediately post-surgery was 2.75 × 10^6^ μm^3^. We compared this to the total volume of regrown eye tissue at st. 42 in development where we found the regrown eye to have an average volume of 8.46 × 10^6^ μm^3^ and where the mean RPC tissue volume taken as a percentage of mean regrown RPC volume was 32.5%.

Wound healing was typically observed 2–4 hours post-surgery, with no apparent rapid dilution of the red RPCs’ Eos concentration relative to their green Eos content (Figure 9, Panels D, D’, D”, E, and E’). This lack of dilution doesn’t imply an absence of red RPC proliferation, but the unchanged dimensions of the post-surgical RPC niche at 2 hps indicate a lack of rapid proliferation at this stage. New cell growth was initially visible at 6 and 8 hps (data not shown), with a small number of cells surrounding the red RPC niche transitioning to a dark orange color, while the majority of the tissue remained red.

By 14 hps, every embryo displayed noticeable orange cells expanding on either side of the initial wedge-shaped red RPC region (Figures 9 and 10, Panels E and E’). These expanding cells formed a tapering crescent whose ends extended, while its opening narrowed. This red portion of the regrowing eye also increased in depth, projecting from the side of the tadpole region (Figures 9 and 10, Panels E-F). The direction of growth was consistent across all tadpoles, regardless of initial RPC quantity, at 26 hps, 38 hps, and 72 hps, as revealed by confocal and multiphoton imaging (Figures 9 and 10). At these time points, the dilution of the label indicated circumferential growth, initiating from the post-surgical RPC cluster and ending at a ventrally located optic fissure filled with yellow-green and light green labeled cells by 72 hps.

Our observations of embryonic eye regrowth are similar and build on the observations of regrowth demonstrated in Kha et al. 2020. In this study the regrowing eye was shown to enlarge from a central region of retinal progenitor cells. The growth as evidenced by increased mitotic activity at 24 hps coincides with our findings of increased eye depth (as measured by confocal z-stack down the depth of the eye) at 26 hps. In addition to the cell growth within the first day post-surgery we observed rapid cell addition to the post-surgical RPC niche at 14 hps while very few new cells were seen at 6 and 8 hps. At 2 hps no additional expansion of the post-surgical RPC niche was observed.

We initially hypothesized that an existing proliferative, RPC niche would facilitate the rapid eye regrowth observed in *Xenopus laevis*. However, the preliminary observation of transdifferentiation in the formation of other eye structures was unexpected. Our data confirm that a post-surgical stem cell niche promotes eye regeneration and may suggest a role for these RPCs in lens regrowth following surgery. Additionally, our preliminary observations may indicate that cells outside the retinal cell lineage, possibly originating from the surrounding epidermis or neural crest, contribute to the regrowth of both the lens and retina. This was initially observed as a bright green labeling of cells in the closing optic fissure for n = 12 of our experimental embryos. These cells represent a population that has gone through the greatest number of cell divisions since the eye removal surgery and was thought to have diluted out their lineage tracer. However, upon analysis with multiphoton imaging, we observed a distinct and abrupt shift from red cells in the regrowing eye to green in the optic fissure and ventral portion of the eye. Based on our estimates, this green region represented about 15% of the regrowing eye.

The dilute red within these cells, in relation to the intense green (Figure 12, Panels A and D), precipitated the need to use another lineage tracing strategy to determine the cell lineage within the optic fissure. We opted to use EosFP, but rather than photoconvert cells in the optic vesicle red, we labeled cells directly outside the green eye red on the ventral half surrounding the eye (Figure 13, Panel A). When we traced these cells to 5 dps, we found them to reside in the ventral portion of the eye in a region consisting of 15% of the eye (Figure 13, Panels E-F), corresponding to the green region when this assay is used to label the retinal cell lineage red. While this is preliminary data, the consistent observation of the green in the optic fissure during a red eye removal surgery, and the red-labeled cells within the optic fissure during a green eye removal surgery, suggest that a cell population outside of the retinal cell lineage is contributing to the regrowth of the eye in this region. While not extensively studied in *Xenopus laevis*, cells in this ventral region during normal eye development in other species are seen to come from the neural crest cell lineage to aid in the formation of the hyaloid artery, ciliary muscles, pupillary muscles, corneal stroma, and the trabecular meshwork, which aids in the aqueous outflow of the anterior chamber of the eye (Whikehart, 2010; Cordero et al., 2011; Kish et al., 2011; Williams & Bohnsack, 2015a).

**Figure 14:**
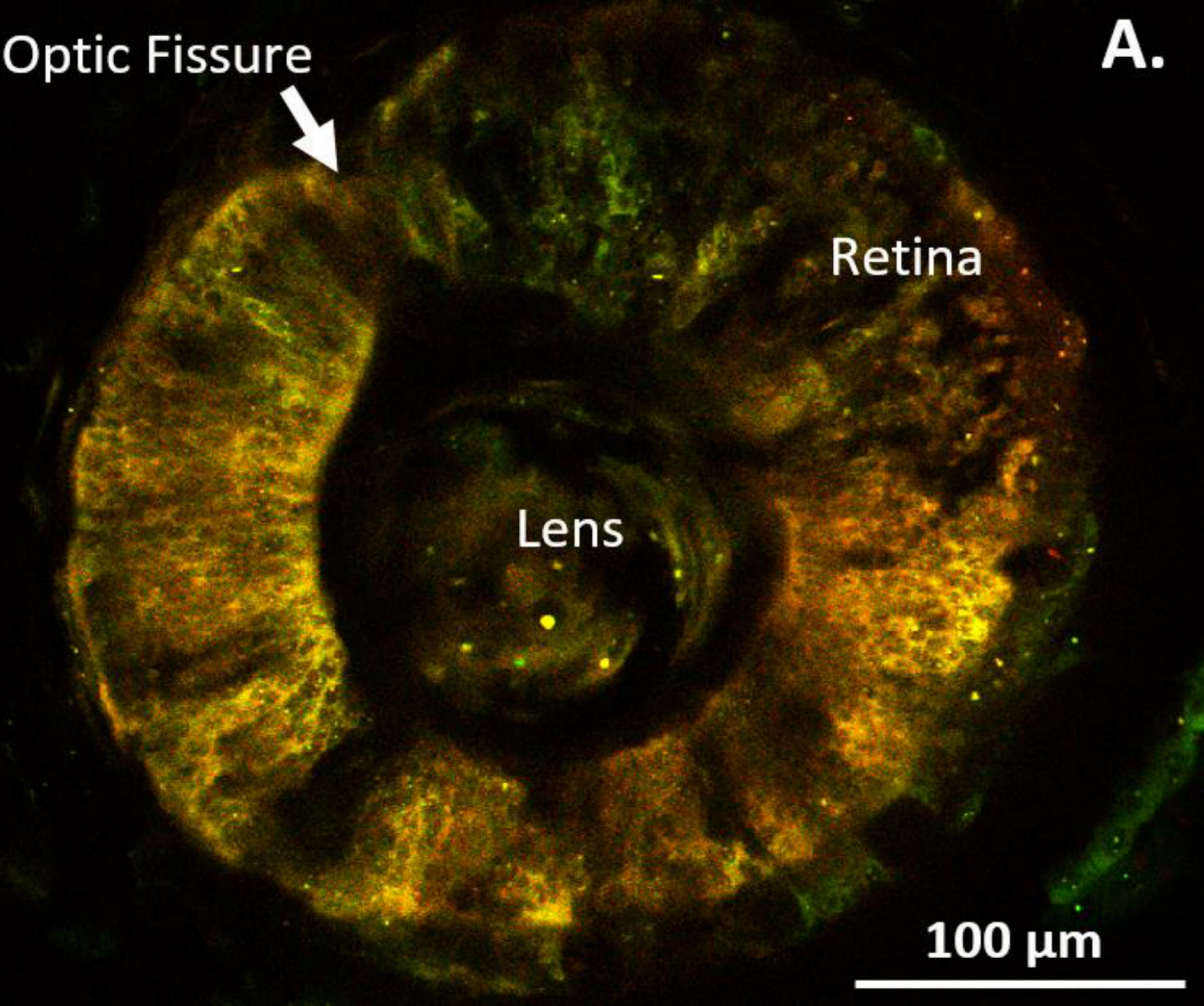
Regrowth of Extraocular Muscles and Retinal Vasculature. Video: https://drive.google.com/file/d/12w5M6iHOqf2-Bj6sAGzOKd4hk8Isa8Sv/view?usp=sharing Panel **(A)** presents an optical section of the red-labeled st. 46 regrowing eye, as depicted in Supplementary Video 1. The ventral portion of the eye is located at the top right-hand side of the image and video. The arrow in Panel A highlights the location of the optic fissure, which remains unclosed. The video demonstrates the movements of the eye during a 20-second sequence captured on a multi-photon microscope at a rate of 1 frame per second (fps). Movements are observed from three sources. An eye saccade, noted at 16 seconds into the video, suggests the functionality of the extraocular muscles and may indicate normal innervation from the cranial nerves: the oculomotor nerve (III), the trochlear nerve (IV), and the abducens nerve (VI). This saccade is akin to typical eye saccades observed in st. 46 tadpoles under light microscopy. Another source of movement is red blood cells traveling through the ophthalmic artery, appearing as unlabeled dark spots moving in a circumferential manner through the eye. A significant movement is observed at the start of the video, likely resulting from a head or tail movement, possibly because the tricaine anesthesia had not fully taken effect.

Endogenous eye growth during development typically involves cells of the non-neural ectoderm overlying the cells of the optic vesicle in close apposition. Reciprocal signaling between these two distinct cell populations prompts the optic vesicle to fold into an optic cup, while the overlying ectoderm assumes a lens fate. However, this reciprocal signaling niche is disrupted during surgery, yet a lens still regrows. Among the tadpoles we observed with a clearly defined lens at stage 41 (n=7), the regenerated lens was comprised of a mixture of orange and green cells (Figure 9, Panels F’ and G’; Figure 11 Panel F; Figure 14, Panel A). Notably, one sample (n=1) had a predominantly green lens (data not shown). The presence of orange-colored cells, resulting from a mix of diluted photoconverted red EosFP and the existing green, unconverted EosFP within the cell, indicates their origin from the post-surgical retinal progenitor cell (RPC) niche. This suggests a contribution to lens tissue formation. Although this labeling might be due to unintended re-photoconversion at stage 18, we expect that cells converted outside the retinal lineage would be eliminated from the eye surface at stage 27 during surgical removal.

To explore this further, we conducted a series of photoconversions on cells of the epidermis surrounding the eye (Figure 13, Panel A). After eye removal (Figure 13, Panel B), we traced these cells during regrowth and discovered their presence in the developing lens (Figure 13, Panel C-E). This observation suggests that the lens can be regrown, at least partially, by cells from the surrounding epidermis. However, the contribution of cells from the RPC lineage to lens regrowth cannot be entirely dismissed. Future research should develop strategies to specifically label the entire epidermal cell lineage around the eye. A limitation of our current labeling method is that only the ventral half of the eye at st. 27 was accessible for labeling, as the mounting angle of the embryo caused the optic vesicle to occlude dorsal epidermal cells. These dorsal cells might contribute more significantly to lens formation than the ventral cells. Nevertheless, this hypothesis does not completely account for the presence of red cells in our RPC labeling strategy. If the ventral epidermis contributes to less than 75% of the lens, this suggests a potential role for other cell populations. Supporting the idea that the lens arises from an epidermal cell lineage, not an RPC lineage, would require findings where labeling the cells above the dorsal half of the st. 27 eye contributes to at least 75% of the lens growth post-surgery. Alternatively, labeling the full epidermis surrounding the eye should result in 100% of the lens being labeled, with no RPC contribution.

An additional source of cells that may be contributing to the regrowth of a mosaic eye could be cells from the optic stalk or cells migrating from the contralateral eye to the ventral region of the regrowing eye. This phenomenon is observed in eye development before embryonic stage 15 (Jacobson and Hirose, 1978) and is evident in our Eos green-labeled embryos as green cells occupying the unlabeled contralateral eye in the ventral anterior region. Similarly, though on rare occasions, it has been observed that red cells from a stage 15 eye field conversion are found in the ventral region of the contralateral eye. While these observations are rare, they do support some of our observations of mosaicism in the ventral region of the unoperated eye. However, even if the stage 27 eye contains some of these mosaic cells that are removed during the eye removal surgery, this population of cells in the optic stalk or ventral anterior region of the contralateral eye may have migrated later than described in the literature. This could potentially be a source of cells that should be tracked in future research in order to eliminate the possibility of non-retinal cells incorporating into the ventral eye and optic fissure.

While our research has illuminated new aspects of the mechanisms driving tadpole eye regrowth, it’s important to acknowledge the limitations of our study and identify avenues for future research. A potential confounding factor in our study was the unintentional labeling of cells outside our primary region of interest, particularly in the olfactory placode, despite our method’s high specificity in targeting cells within the RPC lineage. The incorporation of the red label from these extraneous cell lineages into the regrowing eye could obscure the detection of transdifferentiation from other embryonic cell sources. To address this, we performed imaging at multiple time points and generally did not observe migration from these mislabeled tissues to the wound region, with one notable exception. This particular tadpole exhibited a direct trail of clonally related red cells originating from the olfactory placode and extending into the wound region of the eye (data not shown). Unfortunately, this tadpole did not survive long enough for eye regrowth to be observed, but its case suggests that other cell populations may have the potential to migrate to the wound area during the healing process.

Another aspect to consider is the dilution rate of the photoconverted form of our Eos lineage tracer as cell division occurs. The green form of Eos in the cell dilutes at a slower pace due to the remaining reserve of Eos mRNA being translated within the cell. This resulted in an unexpected benefit: the combined color of the two Eos forms served as an indirect indicator of cell proliferation rate. The slow-dividing RPC population gradually diluted the red to orange color over time, allowing us to track newer eye growth up to developmental stage 42.

However, beyond three days post-surgery, the red Eos signal may become so diluted as to be overshadowed by the stronger green signal, posing challenges in determining cell lineage for any subsequent growth. Therefore, our imaging was confined to the initial three days post-surgery, a period sufficient to observe complete regrowth and the mature eye anatomy. It is important to note that the red form of Eos is sensitive to formaldehyde fixation, which reduces its signal intensity during sectioning at later stages. As a result, we prioritized optical sectioning during live imaging over physical sectioning of the eye. Utilizing multiphoton microscopy was crucial in preserving the faint signal at these later developmental stages, while also providing the benefit of more detailed sectioning along the depth of the eye.

Furthermore, we hypothesized that if the yellow to light green cells observed in the closing optic fissure were a result of red dilution and the changing red:green ratio of Eos within the most developmentally young and proliferative part of the regrowing eye, then labeling cells outside the RPC lineage should not affect the coloring of this eye region with a different labeling strategy. Reflecting on our epidermal cell photoconversion around the ventral half of the eye, we considered whether any of these labeled cells migrated and incorporated into the eye, including the lens. If so, we would expect to see red, clonally related retinal cells within the optic fissure towards the end of the regrowth process. Although our sample size of n=2 is limited, it was surprising to observe red cells forming clonally related columns exclusively within the closing optic fissure after three days of eye regrowth (Figure 13, Panel E). Future research should aim to replicate these findings with a larger sample size while ensuring explicit labeling of only the epidermal cell population. Such research would help to further validate the results observed in this study.

For a comprehensive understanding of the contribution of other cell populations to eye regrowth, future studies should aim to label and trace cells potentially originating from nearby proliferatively active regions. Our lab has noted an increase in cells with mitotic markers after eye removal surgery in the developing brain (unpublished data). Although this region may not contribute massively to eye regrowth, its proximity to the wound area and the ventrally located optic stalk make it a candidate for future lineage tracing. Another vital population for further investigation is the migratory neural crest cells. These multipotent stem cells dissociate from the dorsal part of the neural tube during embryonic development, undergo epithelial-mesenchymal transfer, and migrate to differentiate throughout the length of the embryo (Williams & Bohnsack, 2015a; Pla & Monsoro-Burq, 2018; Szabo & Mayor, 2018). During normal eye development, eye-specific neural crest cells are derived from the multipotent periocular mesenchyme. These migratory mesenchymal cells are instrumental in forming several anterior eye tissues, including the cornea, sclera, and iris. The interactions between these periocular mesenchyme-derived neural crest cells and the optic cup are vital for the morphogenesis of the optic cup and the closure of the optic fissure (Whikehart, 2010; Cordero et al., 2011; Kish et al., 2011; Williams & Bohnsack, 2015a). Our observations suggest that growth in the optic fissure may include contributions from cell lineages beyond the retina. In attempts to label epidermally derived lens cells, we might have inadvertently labeled periocular-derived neural crest cells. This scenario is plausible, given the known integration of this neural stem cell population into the sclera and cornea and their role in optic fissure closure. Differentiating the contributions of epidermal versus neural crest cells is essential for a comprehensive understanding of the mechanisms underlying eye regeneration.

As we draw conclusions from our study, it is important to reiterate the significant findings and implications of our research on *Xenopus* embryonic eye regrowth. We have shown that a post-surgical stem cell niche remains competent and provides the main driving force behind the rapid regeneration we see within the regrowing tadpole eye. We also see initial evidence of a second mode of regeneration, with the lens being regrown from the RPCs switching fates and cell populations potentially from a non-retinal lineage migrating into the eye. Taken in a broader context, this provides further evidence that regeneration is not an identical recapitulation of the processes in development, at least within the *Xenopus* eye regrowth model. Further understanding of the exact contributions of each mode of regeneration will provide a greater mechanistic understanding of the complex interplay of signals and cellular responses required for successful tissue regeneration, and may pave the way for novel regenerative therapies in the future.
